# Chemically induced dimerization of GSDMD C-terminal domain blocks GSDMD N-terminal domain-mediated pyroptosis

**DOI:** 10.1038/s41420-025-02733-0

**Published:** 2025-10-13

**Authors:** Jixuan Xu, Miaoran Fu, Yamin Xing, Wulong Liang, Guangyuan Li, Ting Zhang, Mengxue Li, Chunxiao Gao, Zhanfeng Yang, Yuming Fu, Min Zhang, Zisen Zhang, Pengyuan Zheng, Xiufeng Chu

**Affiliations:** 1 Department of Gastrointestinal & Thyroid Surgery, The Fifth Affiliated Hospital of Zhengzhou University, Zhengzhou, China; 2 Department of Oncology, The Fifth Affiliated Hospital of Zhengzhou University, Zhengzhou, China; 3 Department of Neurology, The Fifth Affiliated Hospital of Zhengzhou University, Zhengzhou, China; 4 Marshall B. J. Medical Center, The Fifth Affiliated Hospital of Zhengzhou University, Zhengzhou, China; 5 Henan International Joint Laboratory of Glioma Metabolism and Microenvironment Research, The Fifth Affiliated Hospital of Zhengzhou University, Zhengzhou University, Zhengzhou, China; 6 Tianjian Laboratory of Advanced Biomedical Sciences, Zhengzhou, Henan China

**Keywords:** Cell death, Cell signalling, Molecular engineering

## Abstract

GSDMD is a pyroptosis executioner in which the C-terminal domain completely inhibits the pore-forming ability of the N-terminal domain. Caspase cleavage separates GSDMD into the free C-terminal fragment (GD-CT) and the free N-terminal fragment (GD-NT). Although GD-CT retains the ability to bind with GD-NT, it can no longer completely disable GD-NT, allowing the latter to oligomerize and form nano-sized pyroptotic pores in the plasma membrane. In this study, we report that GD-CT is strictly confined to the cytoplasm, whereas GD-NT is transported to the plasma membrane. Additionally, GD-CT undergoes rapid degradation via the 26S proteasome pathway. Therefore, we propose that the spatial separation and rapid turnover of GD-CT limit its efficacy in blocking GD-NT-mediated pyroptosis. Given these properties of GD-CT, we engineered an efficient pyroptosis blocker “FKBP-GD-CT”. This chimera protein incorporates a myristoylation motif, which confers plasma membrane translocation capability, and an FKBP12F36V domain, which enables dimerization in response to the chemical inducer AP20187. This is the first report utilizing chemical-induced dimerization technology to modulate pyroptosis levels.

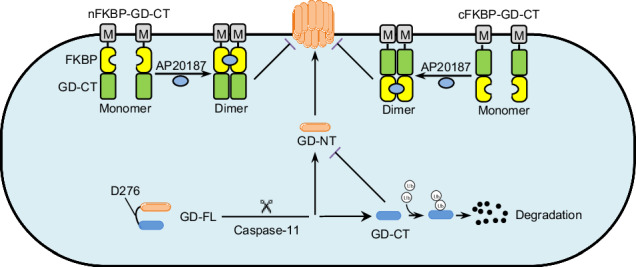

## Introduction

Pyroptosis is a unique form of inflammatory cell death executed by the pore-forming protein “gasdermin” [1]. The human gasdermin family comprises six members: GSDMA, GSDMB, GSDMC, GSDMD, GSDME and DFNB59. Mouse gasdermins are mostly identical to humans, except for the absence of GSDMB and the unique existence of GSDMA1-3 and GSDMC1-4 [2, 3]. To activate the pore-forming activity, gasdermins are recognized and cleaved by appropriate proteases to produce the free gasdermin N-terminal fragment. There are two main branches of the gasdermin activation pathway: canonical and non-canonical [3, 4]. In the canonical pathway, the recognition of pathogen-associated molecular patterns (PAMPs) or damage-associated molecular patterns (DAMPs) by pattern recognition receptors (PRRs) leads to the assembly of inflammasomes, which activate caspase-1 to cleave GSDMD [5]. Several non-canonical pathways are documented. In the presence of cytosolic lipopolysaccharide (LPS), caspase-4/5 (in humans) or caspase-11 (in mice) directly recognizes and cleaves GSDMD [5]. Chemotherapeutic agents, such as etoposide and cisplatin, activate caspase-3 to cleave GSDME in tumor cells [6]. With the aid of perforin, NK cells deliver granzyme A and granzyme B into tumor cells to cleave GSDMB and GSDME, respectively [7, 8]. In some conditions, such as hypoxia, activated caspase-8 also cleaves GSDMC [9]. These liberated gasdermin N-terminal fragments form pores in the plasma membrane and disrupt cellular integrity.

Pyroptosis was first observed in macrophages [10–12] and later in other immune cells and diverse non-immune cells. It not only contributes to defenses against pathogenic organisms [13], but also plays roles in other pathological conditions, such as diabetes [14], chronic inflammation, and cancer [15–18]. Thus, fine-tuning the extent of pyroptosis is valuable for potentially changing the outcome of pyroptosis-related diseases. As the key executioner of pyroptotic cell death, GSDMD has been proven to be a promising pharmacological target for this purpose [19].

To explore the mechanism underlying the low efficiency of GD-CT in blocking GD-NT-mediated pyroptosis, we developed a 293-tetO-GD-NT cell model, in which pyroptosis can be exclusively mediated by GD-NT in response to doxycycline. We found that the spatial separation and rapid turnover of GD-CT are the main factors that compromise its blocking efficacy. To overcome the weakness of GD-CT, we engineered the chimera protein FKBP-GD-CT, the structure and function of which are tuned by AP20187. We found that FKBP-GD-CT combined with AP20187 significantly blocked GD-NT-mediated pyroptosis.

## Results

### The pyroptosis model cell line 293-tetO-GD-NT is created to assess the blocking ability of GD-CT

Pyroptosis signaling pathways converge on the activation of specific proteases, which liberate the cytolytic gasdermin *N*-terminal domain from the inhibitory gasdermin C-terminal domain (Extended Fig. 1A, B). For the first identified pyroptosis executioner GSDMD, the separation of the *N*-terminal domain and C-terminal domain is achieved via caspase-1/11-mediated cleavage, allowing the manifestation of its pore-forming activity (Extended Fig. 1C, D). Interestingly, despite binding and inhibiting GD-NT after cleavage [5], GD-CT can no longer prevent the occurrence of pyroptosis. This phenomenon implies the low efficiency of GD-CT in blocking GD-NT-mediated pyroptosis.

**Fig. 1:**
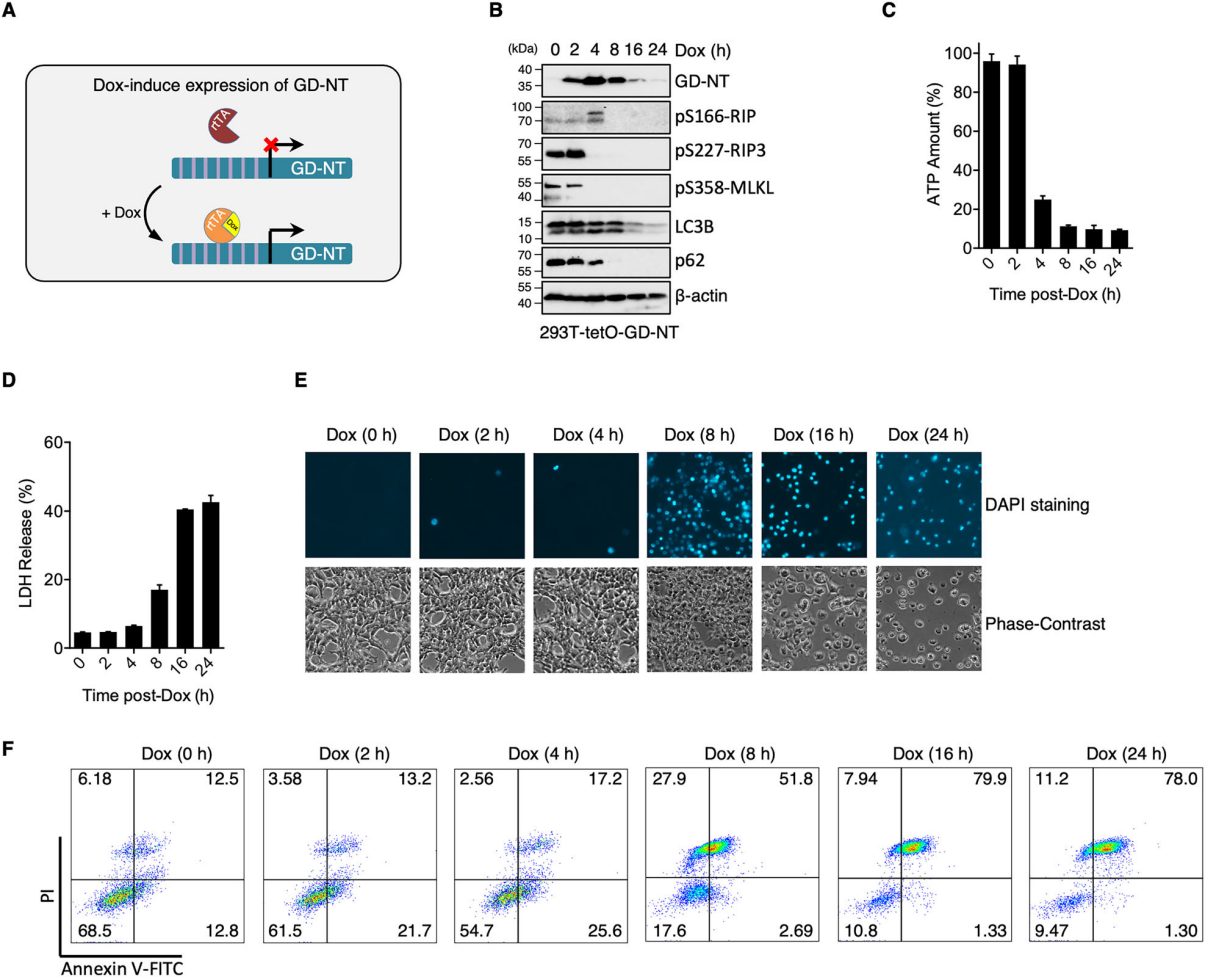
Pyroptosis cell model 293-tetO-GD-NT is set up to study the regulation mechanisms of pyroptosis. **A** Tetracycline on (TetOn) system uses tetracycline (or one of its analogs like doxycycline) as a regulator of gene expression. Tetracycline-dependent promoter is created by placing a TRE upstream of a minimal promoter. TRE is 7 repeats of tetracycline operator (tetO) sequence and is recognized by reverse tetracycline-controlled transactivator (rtTA). In the presence of tetracycline or one of its analogs like doxycycline (Dox), rtTA binds to the TRE and tetracycline, permitting transcription. To create 293-tetO-GD-NT cells, HEK293 cells were transduced with lentivirus to express Dox-inducible GD-NT. **B** Immunoblot (IB) analysis of 293-tetO-GD-NT cells. The cells were treated with Dox (2 µg/mL) and harvested at different time points to obtain whole cell lysate (WCL). IB analysis of WCL was performed with different antibodies. β-actin served as an internal control. **C** Similar to (**B**), except that the cells were subjected to ATP-based Cell Viability Assay. **D** Similar to (**B**), except that the cells were subjected to LDH-based Cytotoxicity Assay. **E** Similar to (**B**), except that the morphological changes were observed using phase-contrast imaging (upper panel). Dying cells were stained with DAPI for fluorescent microscope imaging (lower panel). **F** Similar to (**B**), except that cell death was analyzed by flow cytometry using Annexin V/PI staining kit. In **C** and **D**, differences among groups were analyzed by two-tailed Student’s t-test (means ± s.e.m). Error bars represent the variation range of duplicated experiments. Data are representative of at least two independent experiments.

To explore the biological characteristics of GD-CT underlying its limited blocking efficacy, we created a pyroptosis model cell line “293-tetO-GD-NT”, in which GD-NT expression is induced by Dox (Fig. 1A). Immunoblot showed rapid GD-NT expression, detectable at 2 h and peaking at 4 h post-Dox (Fig. 1B). Significant ATP decline occurred by 4 h (Fig. 1C), while LDH release was evident at 8 h (Fig. 1D). DAPI staining revealed obvious cell death at 8 h, with most cells dead by 24 h (Fig. 1E). Cell death was quantitatively analyzed using Annexin V-FITC/PI double-staining kit [20–23]. Flow cytometry revealed that 293-tetO-GD-NT underwent progressive pyroptosis over time (Fig. 1F). These data demonstrate the Dox-inducible pyroptosis in 293-tetO-GD-NT and suggest it is a suitable model for assessing GD-CT blockade.

### GD-CT blocks pyroptosis in a dose-dependent but inefficient manner

**Fig. 2:**
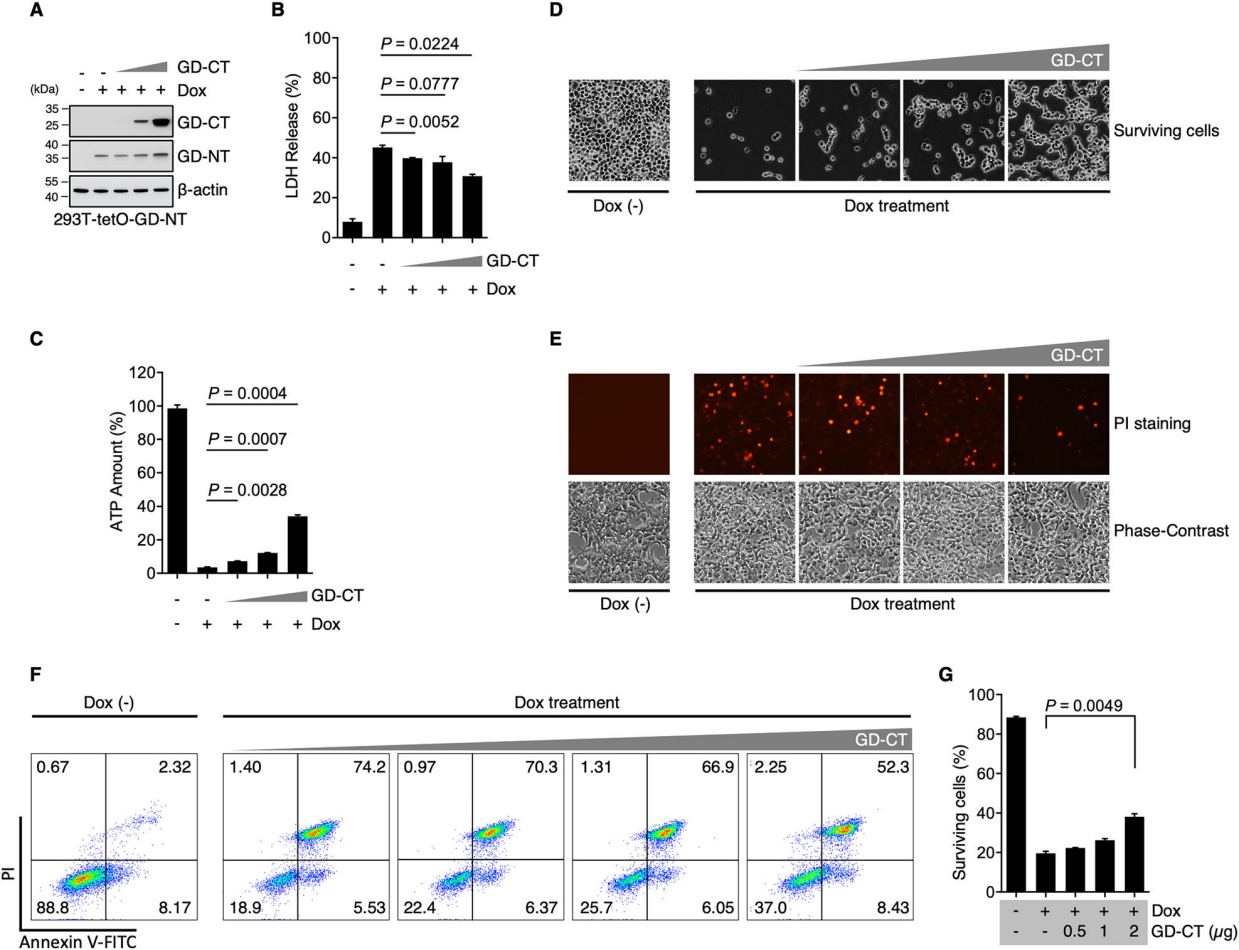
GD-CT blocks pyroptosis in a dose-dependent but inefficient manner. **A** 293T-tetO-GD cells were transfected with different doses of GD-CT. 48 hours post-transfection, the cells were treated with Dox (2 µg/mL) to induce GD-NT expression. 4 h post-Dox, the cells were harvested for IB analysis with indicated antibody. **B** Similar to (**A**), except that the cells were harvested 24 h post-Dox and subjected to LDH-based Cytotoxicity Assay. **C** Similar to (**A**), except that the cells were harvested 24 h post-Dox and subjected to ATP-based Cell Viability Assay. **D** Similar to (**A**), except that, 24 h post-Dox, the cells floating in the culture medium were removed, and the adherent cells (considered as living cells) were subjected to phase-contrast imaging. **E** Similar to (**A**), except that, 4 h post-Dox, the cells were stained with PI and analyzed under immunofluorescence microscope. PI-positive cells were considered pyroptotic cells in this context. **F**. Similar to (**A**), except that, 14 h post-Dox, cell death in 293-tetO-GD was analyzed via flow cytometry and Annexin V/PI staining kit. **G** The proportion of surviving cells in F was statistically analyzed. In (**B**, **C**, and **G**,) differences among groups were analyzed by two-tailed Student’s t-test (means ± s.e.m). Error bars represent the variation range of duplicated experiments. Data are representative of at least two independent experiments.

First, we transiently transfected empty vector (EV) or GD-CT into 293-tetO-GD-NT cells and verified their expression by immunoblotting (Fig. 2A). To assess GD-CT’s blocking ability, Dox was added after transfection to induce the expression of GD-NT and subsequent pyroptosis. 14 hours post-Dox, cells and culture media were collected for Cell cytotoxicity/viability assays, phase-contrast and fluorescence microscopy, and flow cytometry analysis. Our results showed that GD-CT reduced LDH release and restored ATP production in a dose-dependent manner (Fig. 2B, C). Consistently, GD-CT significantly increased cell viability (Fig. 2D, E). Compared with the ~20% survival rate in the EV-control group, GD-CT dose-dependently increased survival to a maximum of ~40% (Fig. 2F, G). These findings indicate that GD-CT incompletely but dose-dependently blocks pyroptosis.

### GD-CT blocks GD-NT in a unique way distinct from its precursor “C-terminal domain of GSDMD”

**Fig. 3:**
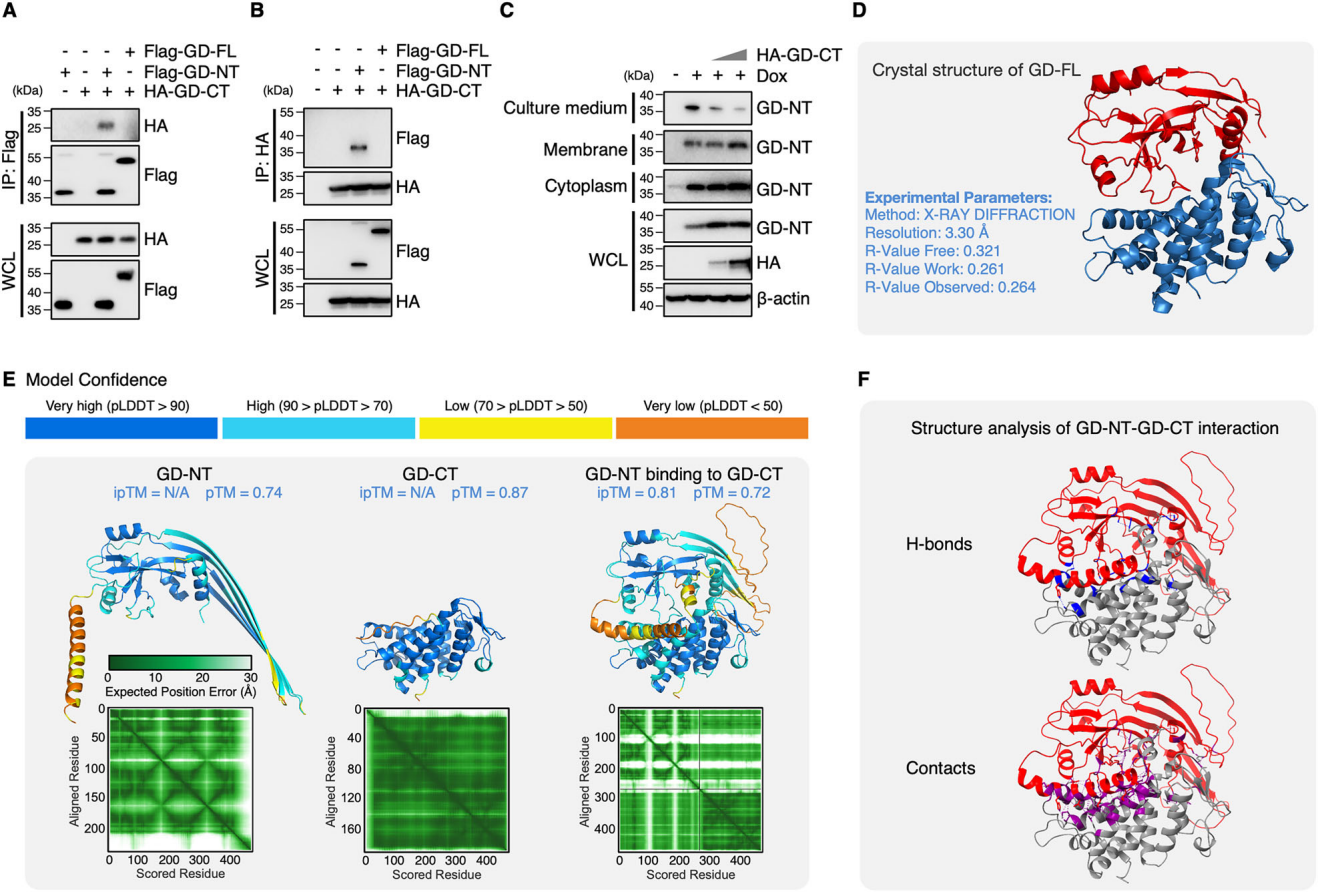
GD-CT blocks GD-NT in a unique way distinct from its precursor “C-terminal domain of GSDMD”. **A**, **B** HEK293 cells were co-transfected with Flag-GD-FL, Flag-GD-NT and HA-GD-CT. 16 h later, the cells were harvested for anti-Flag (A) or anti-HA (B) IP assay. The precipitants and WCL were immunoblotted with indicated antibodies. **C** Subcellular localization of GD-NT and GD-CT. 293T-tetO-GD cells were transfected with different doses of GD-CT. 48 h post-transfection, the cells were treated with Dox (2 µg/mL) to induce GD-NT expression. 16 h post-Dox, the cells were fractionated separately. Proteins in culture medium, cytoplasm, membrane, or WCL were subjected to IB analysis with the indicated antibodies. StrataClean beads were used to precipitate and enrich proteins from the culture medium. **D** Experimentally determined murine GSDMD structure (Method X-RAY DIFFRACTION; Resolution 3.30 Å) was downloaded from RCSB PDB (PDB code: 6N9N) and visualized via PyMOL. **E** The structures of GD-FL, NT, and CT, the interaction between the free GD-NT and the free GD-CT protein were predicted via AlphaFold3 and then visualized via PyMOL (3D structure) and ChimeraX (PAE plot). N/A: not applicable. **F** Structure Analysis via ChimeraX. Hydrogen bonds (H-bonds, Blue) and contacts (Purple) were identified between the free GD-NT (Red) and the free GD-CT (Gray). Data are representative of at least two independent experiments.

Interestingly, despite interacting with GD-NT and inhibiting its release into the culture medium (Fig. 2F, G, and Fig. 3A–C), GD-CT failed to block pyroptosis as efficiently as the intramolecular inhibition mechanism within full-length GSDMD (GD-FL), in which the C-terminal domain completely blocks the pore-forming ability of the N-terminal domain and renders GD-FL noncytolytic. We hypothesized that the low efficiency of GD-CT arises from structural changes induced by proteolytic cleavage at the interdomain linker region of GD-FL. To test this hypothesis, 3D structural prediction was performed using AlphaFold 3 [24]. The predicted 3D structures were visualized using PyMOL and ChimeraX. We found that the structure of the GD-CT-GD-NT complex was conformationally distinct from that of GD-FL (Fig. 3D–F). These structural differences may explain the divergences in their biological activities and, consequently, blocking efficiency in GD-NT-mediated pyroptosis. Next, we conducted experiments to investigate the molecular properties of GD-CT, including its subcellular localization and protein stability.

### The restriction of GD-CT to the cytoplasm causes spatial isolation

**Fig. 4:**
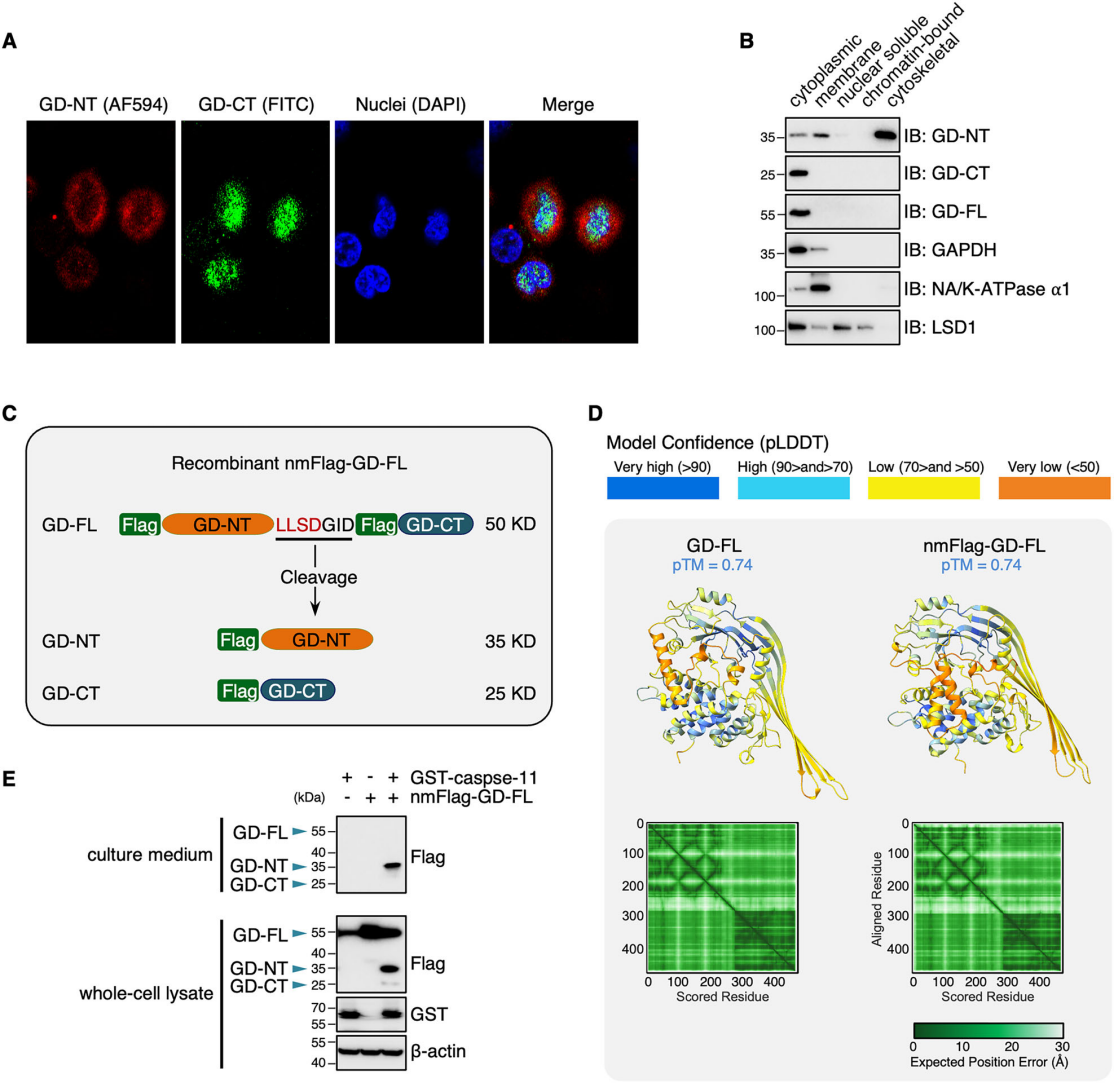
The restriction of GD-CT in the cytoplasm causes spatial isolation. **A** Different distribution of NT and CT in cells. HeLa cells were co-transfected with Flag-GD-NT and HA-GD-CT. 18 hours later, the cells were fixed with methanol, stained with anti-Flag antibody conjugated to Alexa Fluor 594 (AF594, red) and anti-HA antibody conjugated to FITC (green), and counterstained with Hoechst. Subcellular localization of GD-NT and GD-CT was analyzed by confocal microscopy. **B** HEK293 cells were transfected with the indicated constructs. 16 h later, the cells were harvested and subjected to sample preparation using the Thermo Fisher Subcellular Fractionation Kit. Different fractions were subjected to IB analysis with the indicated antibodies. GAPDH, NA/K-ATPase α1, and LSD1 served as internal controls. **C** Design of nmFlag-GD-FL. In the schematic illustrative cartoon, GSDMD is Flag-tagged on both N-terminal of GD-NT domain and N-terminal domain of GD-CT domain. Cleavage of nmFlag-GD-FL by caspase-1/11 produces nFlag-GD-NT and nFlag-GD-CT at a ratio of 1:1. This allows the simultaneous detection of GD-NT, GD-CT with a unique Flag-antibody. **D** The structures of GD-FL, nmFlag-GD-FL were predicted via AlphaFold3 and then visualized via PyMOL (3D structure) and ChimeraX (PAE plot). N/A: not applicable. **E** HEK293 cells were transfected with nmFlag-GD-FL and/or GST-caspase-11 GD-NT. 24 h later, the proteins in cells and culture media were harvested, respectively, and subjected to immunoblot analysis with indicated antibodies. Data are representative of at least two independent experiments.

To assess the subcellular localization of GD-CT, we co-transfected Flag-tagged GD-NT and HA-tagged GD-CT into HeLa cells and performed confocal microscopy. It showed that GD-CT remained in the cytoplasm, and GD-NT was primarily translocated to the plasma membrane (Fig. 4A and Extended Fig. 2). To precisely determine their distribution, we transfected these GSDMD constructs into HEK293 cells and subjected them to subcellular fractionation, which separates cellular components into five compartments: soluble cytoplasmic, membrane, soluble nuclear, chromatin-bound nuclear, and insoluble cytoskeletal. Immunoblot analysis revealed that GD-CT was exclusively localized in the cytoplasm, whereas GD-NT was detected in the cytoplasm, plasma membrane, and cytoskeleton (Fig. 4B).

To imitate the dynamic changes of GSDMD localization during caspase-11 cleavage, we engineered a construct, “nmFlag-GD-FL” (Fig. 4C), with an N-terminal Flag-tag on both GD-NT and GD-CT for simultaneous detection using the same antibody. 3D structural prediction with AlphaFold 3 confirmed that the Flag-tags did not artificially disrupt GSDMD structure (Fig. 4D). We then transfected HEK293 cells with nmFlag-GD-FL and caspase-11 and separately harvested proteins from cells and culture medium. Immunoblot analysis showed that significantly less GD-CT was retained in cells, and no GD-CT was detected in culture medium. (Fig. 4E). Thus, our findings indicate that spatial isolation underlies the inefficiency of GD-CT in blocking GD-NT-mediated pyroptosis: The cytoplasmic GD-NT is bound and inhibited by co-localized GD-CT, while the GD-NT in the plasma membrane or culture medium remains active.

### The short half-life of GD-CT contributes to the liberation of GD-NT

**Fig. 5:**
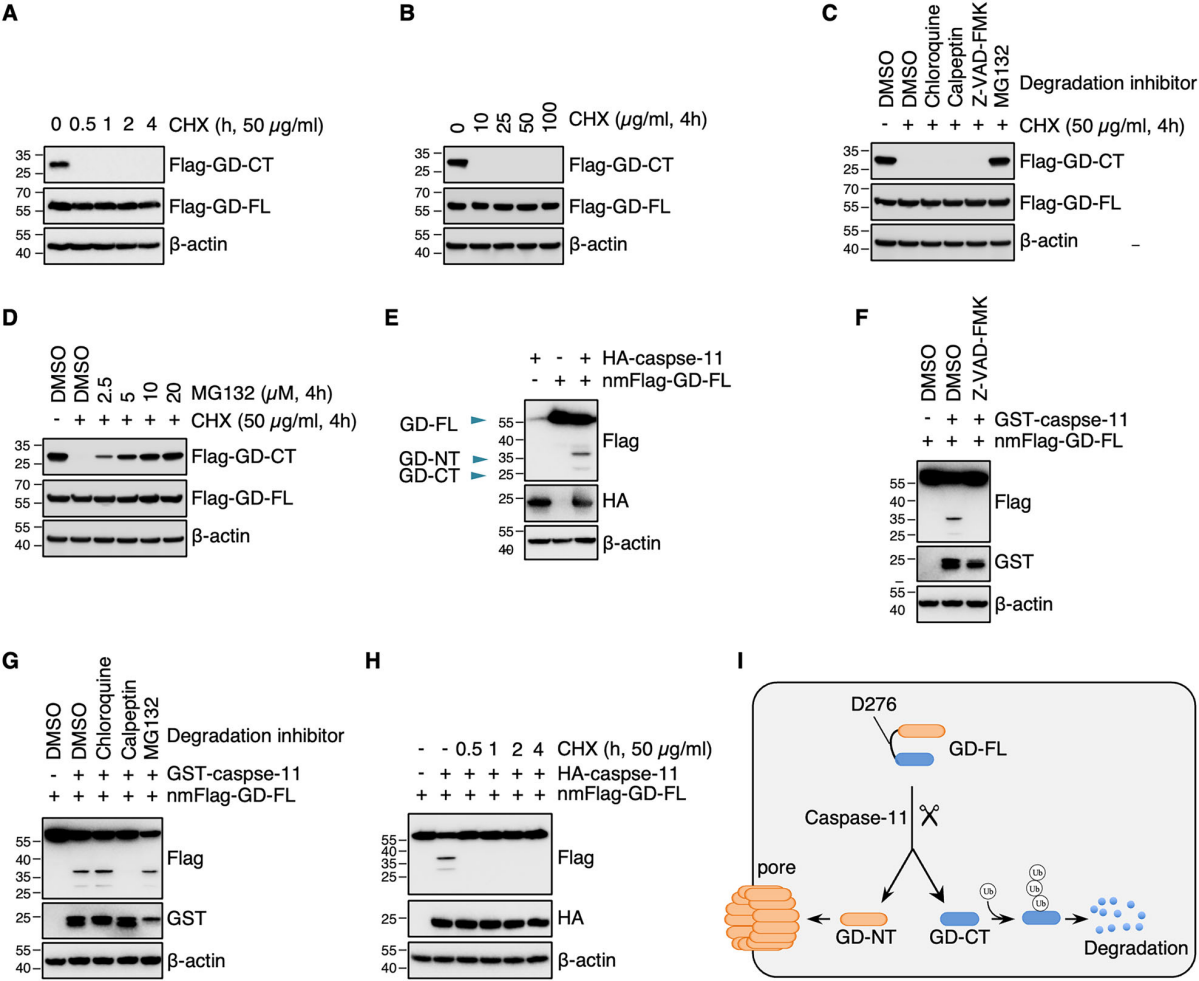
The short half-life of GD-CT contributes to the liberation of GD-NT. **A** HEK293 cells were transfected with Flag-GD-FL, GD-NT, or GD-CT, respectively. 16 h later, CHX (50 µg/mL) was used to block protein synthesis. The cells were harvested at different time points post-CHX and subjected to IB analysis with the indicated antibodies. *β*-actin served as an internal control. **B** Similar to (**A**), except that different concentrations of CHX were used to block protein synthesis, and the cells were harvested at 4 h post-CHX. C. Similar to (**A**), except that the cells were treated with CHX (50 µg/mL) and different protease inhibitors including Chloroquine (100 µM), Calpeptin (50 µM), Z-VAD-FMK (50 µM), and MG132 (20 µM). **D** Similar to (**C**), except that the cells were treated with CHX (50 µg/mL), and different concentrations of MG132 were used. **E** HEK293 cells were transfected with nmFlag-GD-FL and/or HA-caspase-11. 24 h later, the cells were harvested for IB analysis with the indicated antibodies. **F**,**G** HEK293 cells were transfected with nmFlag-GD-FL and/or GST-caspase-11, and, at the same time, treated with one of the following chemicals to block the activities of diverse proteases: Chloroquine (100 µM), Calpeptin (50 µM), Z-VAD-FMK (50 µM), MG132 (20 µM). 24 hours later, the cells were harvested for IB analysis with the indicated antibodies. **H** HEK293 cells were transfected with nmFlag-GD-FL and/or HA-caspase-11. 20 h later, CHX (50 µg/mL) was used to block protein synthesis. The cells were harvested at different time points post-CHX and subjected to IB analysis with the indicated antibodies. **I** Schematic illustration of GD-CT degradation. When cut by caspase-11, GD-FL produces GD-NT and GD-CT. The inhibitory GD-CT undergoes a fast 26S protease-dependent degradation; thus, the redundant cytolytic GD-NTs form pores on the plasma membrane and cause pyroptosis. Data are representative of at least two independent experiments.

To assess the stability of GD-CT, HEK293 cells were transfected with Flag-GD-CT or Flag-GD-FL and then treated with CHX, a commonly used protein synthesis inhibitor. We found that GD-CT had a much shorter half-life and became undetectable within 4 h of CHX treatment, whereas GD-FL showed no degradation during the observation period (Fig. 5A, B). To identify its degradation pathways, we used several inhibitors to target specific processes: chloroquine (autophagy), calpeptin (calpain activity), Z-VAD-FMK (caspase activity), and MG132 (proteasome pathway). Our results showed that only MG132 completely blocked GD-CT degradation, indicating rapid turnover via the ubiquitin-proteasome system (Fig. 5C, D).

As shown in Fig. 4C, the construct nmFlag-GD-FL enables the simultaneous assessment of GD-CT and GD-NT turnover using the same antibody, making it an ideal model for the concurrent comparative evaluation. Since cleavage occurs at a specific site, GD-CT and GD-NT should theoretically be produced in equimolar amounts (in a 1:1 ratio). Surprisingly, GD-CT levels were significantly lower than GD-NT in cells (Fig. 5E–H). Therefore, we conclude that GD-FL is stable, but upon caspase-11 cleavage, the produced GD-CT undergoes faster degradation than GD-NT, reducing its capacity to block GD-NT-mediated pyroptosis (Fig. 5I).

### The design of the chimera protein FKBP-GD-CT

Blocking pathological pyroptosis, such as in sepsis, holds significant clinical importance, making the exploration of pyroptosis inhibitors an active research focus. GD-CT is a natural inhibitor of GD-NT-mediated pyroptosis, but its low efficiency hinders its potential in biomedical research and clinical applications. Given that GD-CT’s inefficiency results from its cytosolic retention and rapid degradation, we tried to engineer a chimeric GD-CT to address these limitations.

**Fig. 6:**
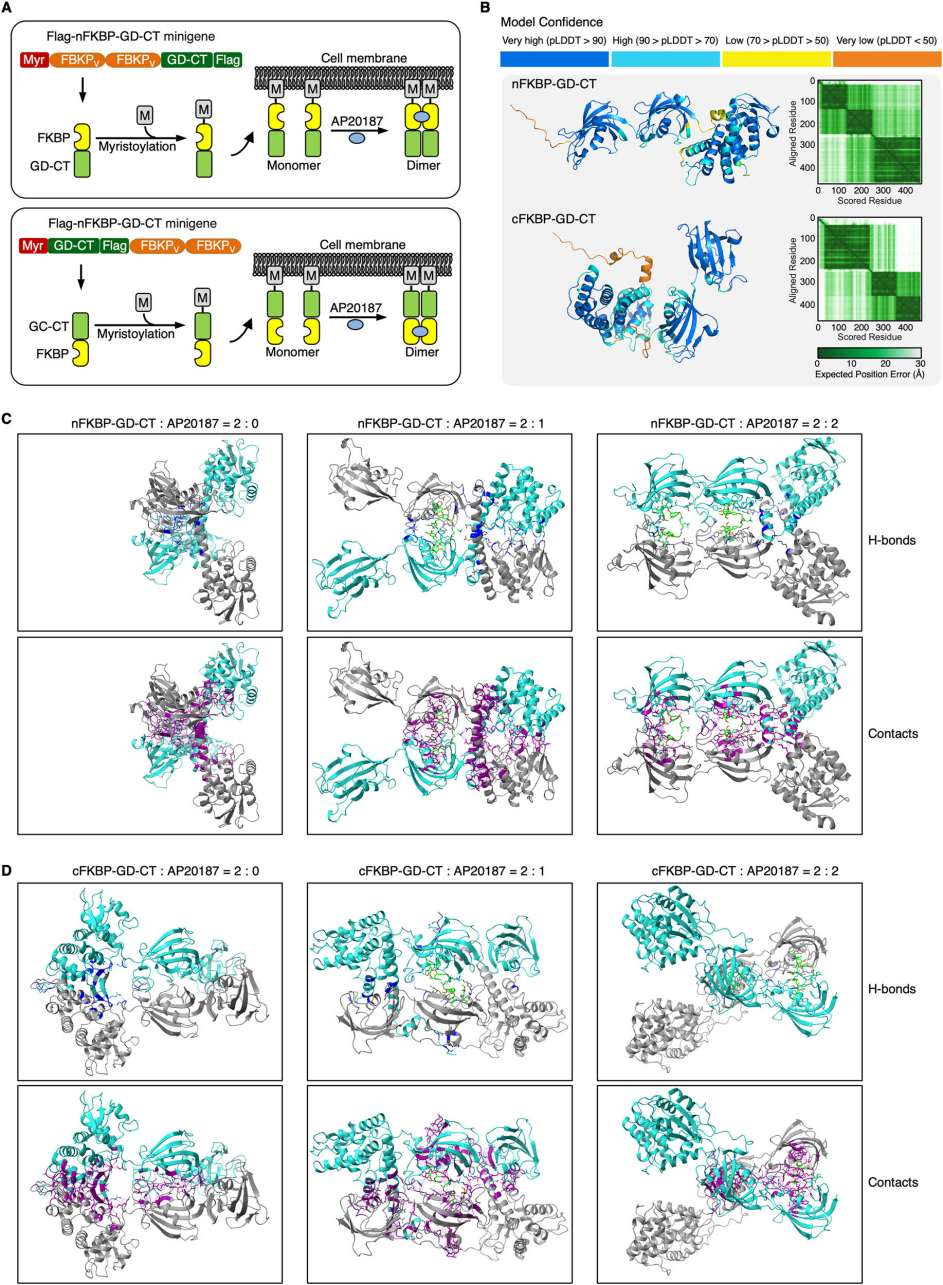
The design of chimera protein FKBP-GD-CTs. **A** Schematic illustration of the design of the chimera protein FKBP-GD-CTs. **B** The structure of FKBP-GD-CTs was predicted via AlphaFold3 and then visualized via PyMOL (3D structure) and ChimeraX (PAE plot). N/A: not applicable. **C**, **D** The interaction of AP20187 and FKBP-GD-CTs was predicted via AlphaFold3 and then visualized via PyMOL (3D structure) and ChimeraX (PAE plot). N/A: not applicable. Hydrogen bonds (H-bonds, blue) and contacts (purple) were identified between the AP20187 (green) and the free GD-CT (gray or marine).

First, GD-NT oligomerizes and forms pores at the plasma membrane, membrane targeting is essential to enable the engineered chimera to directly interact with membrane-bound GD-NT. Myristoylation (Myr) is a vital lipid modification that facilitates protein targeting to membranes [25]. Based on vertical-scanning of sequence requirements for *N*-myristoylation [26], we fused a common Myr motif “GSSKSKPKDPSQR” to GD-CT to confer membrane translocation capability. Second, dimerization significantly influences protein conformation and stability [27–30]. For instance, cytosolic disulfide bridge-supported dimerization is crucial for the stability and cellular distribution of Coxsackievirus B3 protein 3 A [31]. FK506 binding protein 12 (FKBP12) is typically monomeric but dimerizes in the presence of ligands such as FK506 (for FKBP12^WT^) and AP20187 (for FKBP12^F36V^) [32]. As genetically engineering of proteins using this chemically induced dimerization (CID) system enables manipulation of protein localization, signaling pathways, and activation [33–38], we fused two copies of FKBP12^F36V^ to GD-CT to confer a prolonged half-life upon AP20187 treatment. These designs yielded the chimera protein FKBP-GD-CT, including nFKBP-GD-CT and cFKBP-GD-CT (Fig. 6A and Extended Table 1). We first used AlphaFold 3 and PyMOL to predict and visualize the 3D structure of nFKBP-GD-CT and cFKBP-GD-CT (Fig. 6B), followed by Chai Discovery and ChimeraX to analyze the interaction between FKBP-GD-CT and AP20187, including hydrogen (H) bonds and other contacts. We found that AP20187 binding induced distinct conformational changes in both nFKBP-GD-CT and cFKBP-GD-CT (Fig. 6C, D).

### Biological characterization of the chimera protein FKBP-GD-CT

**Fig. 7:**
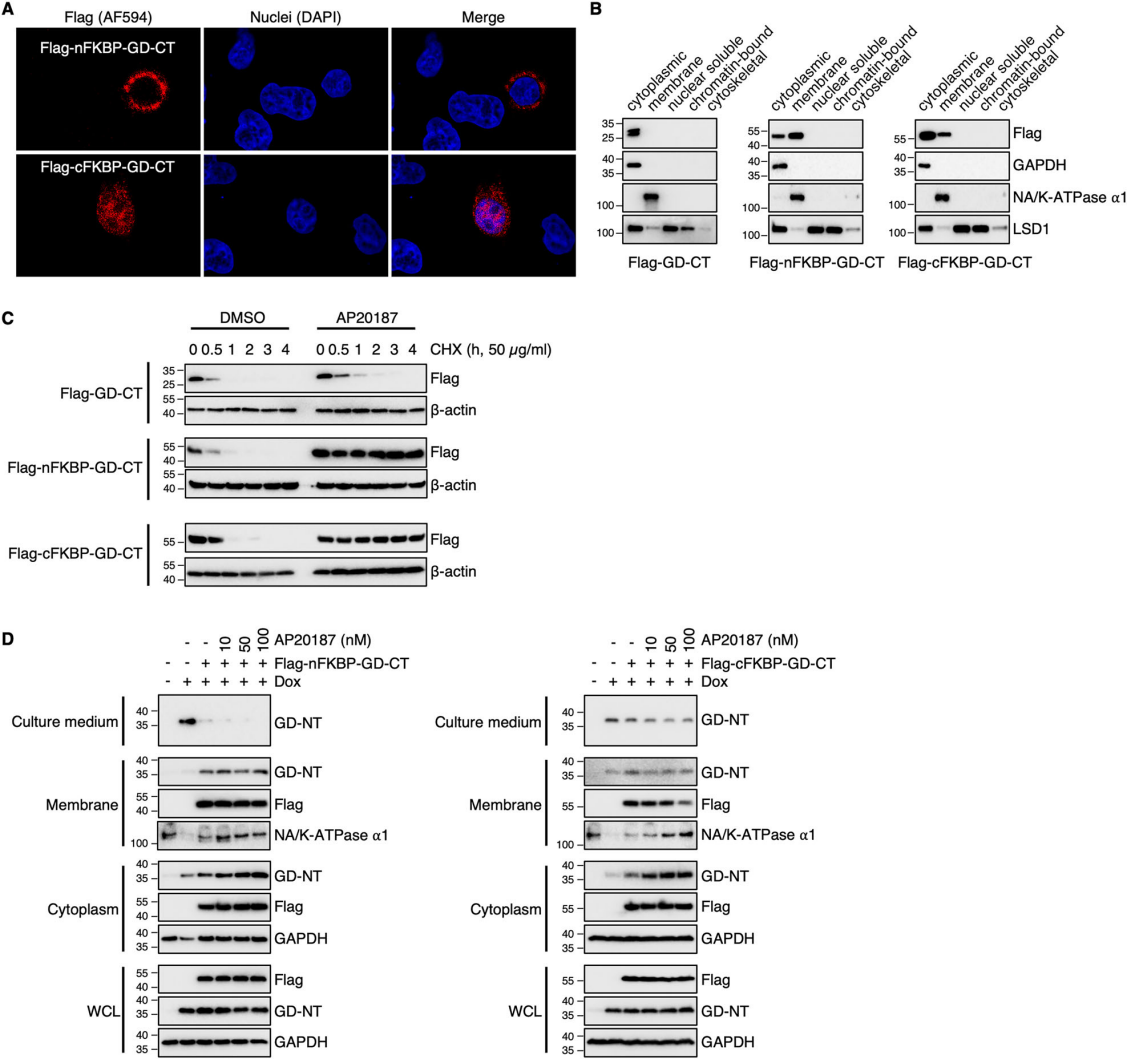
The biological characteristics of chimera protein FKBP-GD-CTs. **A** Distribution of FKBP-GD-CT by fluorescence microscopy. HeLa cells were transfected with Flag-FKBP-GD-CT individually. 18 h later, the cells were fixed with methanol, stained with mouse FITC-Flag antibody, and counterstained with DAPI. The distributions of these proteins were visualized by fluorescence microscopy. **B** HEK293 cells were transfected with the indicated constructs. 16 h later, the cells were harvested and subjected to sample preparation using the Thermo Fisher Subcellular Fractionation Kit. Different fractions were subjected to IB analysis with the indicated antibodies. GAPDH, NA/K-ATPase α1, and LSD1 served as internal controls. **C** HEK293 cells were transfected with the indicated constructs. 16 hours later, the cells were treated with CHX (50 µg/mL) and/or AP20187 (100 nM) and then harvested at different time points for IB analysis with the indicated antibodies. **D** 293-tetO-GD-NT cells were transfected with the indicated constructs. Different concentrations of AP20187 were added at the same time as transfection. 14 h later, Dox (2 µg/mL) was added to induce the expression of GD-NT. The cells were harvested at 8 h post-Dox for subcellular fractionation. The proteins in culture medium, membrane, cytoplasm, as well as WCL were subjected to IB analysis with the indicated antibodies. StrataClean beads were used to precipitate and enrich proteins from the culture medium. Data are representative of at least two independent experiments.

To assess the subcellular localization of FKBP-GD-CT, we transfected HEK293 cells with nFKBP-GD-CT or cFKBP-GD-CT and performed confocal microscopy and subcellular fractionation analyses. Our results revealed that nFKBP-GD-CT or cFKBP-GD-CT localized to both the cytoplasm and the plasma membrane (Fig. 7A, B), indicating that the Myr motif successfully facilitates their membrane targeting. Next, to assess FKBP-GD-CT stability, HEK293 cells transfected with these constructs were treated with CHX and/or AP20187, followed by immunoblot. We found that AP20187 treatment significantly extended the half-life of nFKBP-GD-CT or cFKBP-GD-CT. (Fig. 7C). Finally, to test whether FKBP-GD-CT inhibits the translocation of GD-NT to the plasma membrane or its release into culture medium, HEK293 cells transfected with these constructs were subjected to subcellular fractionation and immunoblot. The data showed that nFKBP-GD-CT or cFKBP-GD-CT reduced GD-NT release into the culture medium, and AP20187 further enhanced this inhibitory effect (Fig. 7D).

### FKBP-GD-CT blocks GD-NT-mediated pyroptosis in a more efficient way

**Fig. 8:**
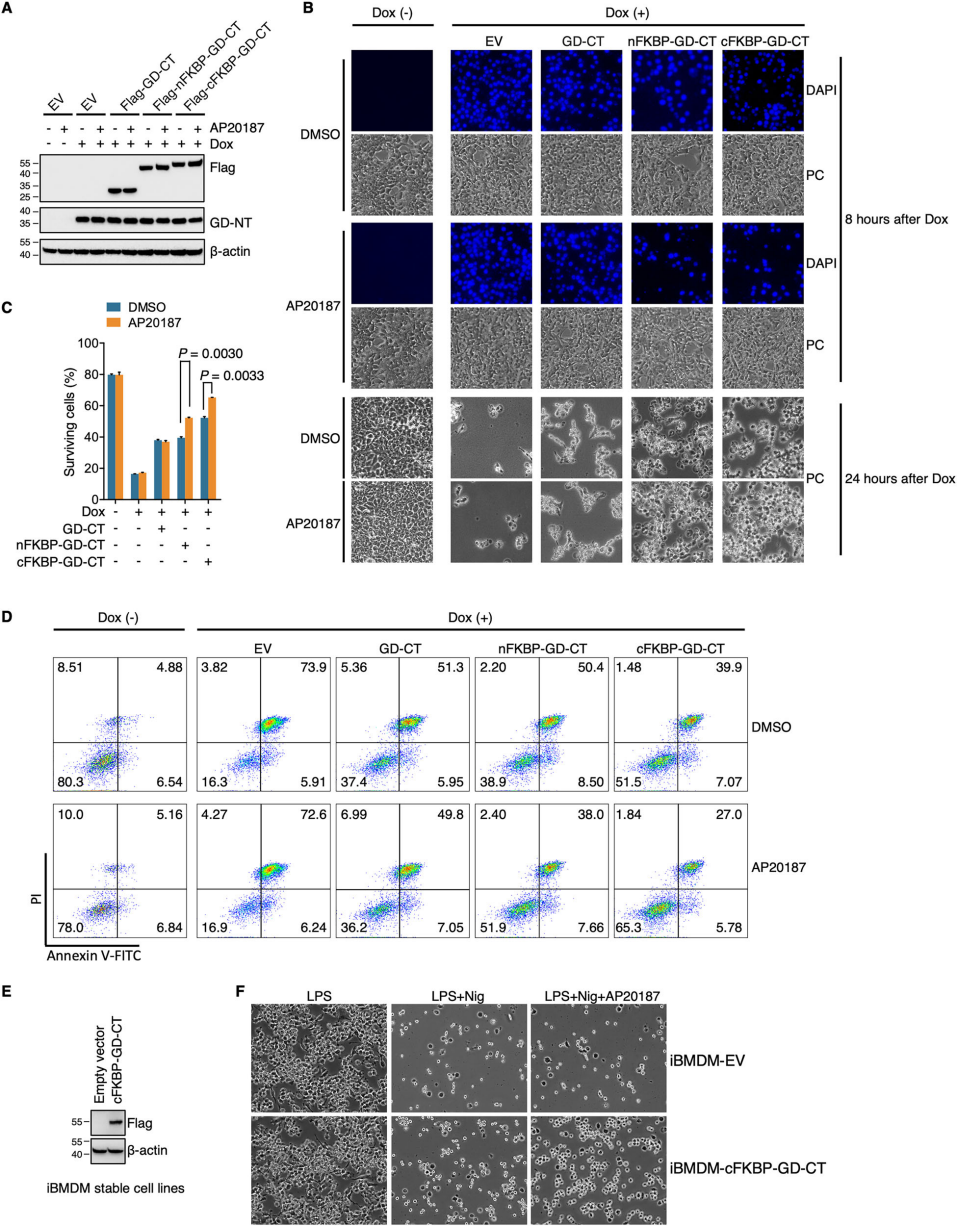
FKBP-GD-CTs block GD-NT-mediated pyroptosis in a more efficient way. **A** 293-tetO-GD-NT cells were transfected with the indicated constructs. 24 h later, the cells were treated with Dox (2 µg/mL) and/or AP20187 (100 nM). 16 h after Dox treatment, the cells were subjected to IB analysis with the indicated antibodies. **B** Similar to (**A**), except that, 8 h after Dox treatment, the cells were stained with DAPI (1 µg/mL) and imaged under immunofluorescence microscope. DAPI-positive cells were considered pyroptotic cells in this context. 24 h after Dox treatment, the cells floating in culture medium were removed, and the adherent cells (considered as living cells) were subjected to phase-contrast imaging. **C**,**D** Similar to (**A**), except that 14 h after Dox treatment, the cells were analyzed by flow cytometry using the Annexin V/PI staining kit. Shown are the proportion analysis of surviving cells (C)and the dot plot (D). Note: Annexin-V/PI double-negative cells were defined as surviving cells. **E** iBMDM cells were constructed to stably express EV (iBMDM-EV) or Flag-nFKBP-GD-CT (iBMDM-cFKBP-GD-CT). The expression of cFKBP-GD-CT was confirmed through IB analysis with the indicated antibodies. **F** iBMDM-EV or iBMDM-cFKBP-GD-CT were treated with LPS (1 µg/mL, 6 h) followed by Nigericin (2 µM, 3 h). AP20187 (1 µM) was added at the same time as LPS. The floated cells were removed, and the adherent cells (considered as living cells) were subjected to phase-contrast imaging. In C, differences among groups were analyzed by two-tailed Student’s t-test (means ± s.e.m). Error bars represent the variation range of duplicated experiments. Data are representative of at least two independent experiments.

To evaluate the inhibitory capacity of FKBP-GD-CT against GD-NT-mediated pyroptosis, we employed two cell models: the 293-tetO-GD-NT cells and the murine macrophage cell line iBMDM. In the 293-tetO-GD-NT model, the cells were transfected with the original GD-CT or the chimeric FKBP-GD-CT. After verifying their expression via Immunoblot (Fig. 8A), pyroptosis was assessed using two approaches: First, the cells without fixation were stained with DAPI and subjected to fluorescence microscopy, in which the DAPI-positive cells represent dying cells. The results showed that FKBP-GD-CT significantly reduced dying cells, an effect further enhanced by AP20187 (Fig. 8B). Next, flow cytometry was performed to quantify the dying cells, which revealed less pyroptosis in the cells transfected with FKBP-GD-CT compared to the original GD-CT, with AP20187 amplifying this reduction. Notably, cFKBP-GD-CT showed superior inhibitory efficacy to nFKBP-GD-CT (Fig. 8C, D).

In iBMDM cells, pyroptosis can be triggered with LPS + Nigericin (Nig), allowing evaluation of FKBP-GD-CT’s blocking efficacy in a relatively physiological setting. We generated stable cell lines expressing cFKBP-GD-CT or empty vector control via lentiviral transduction (Fig. 8E). These cells were pretreated with LPS and/or AP20187 for 6 h, then treated with Nigericin for 3 h. Consistent with the findings obtained from 293-tetO-GD-NT, AP20187 substantially enhanced cFKBP-GD-CT’s inhibition of pyroptosis in iBMDM cells (Fig. 8F). Thus, the engineered FKBP-GD-CT colocalizes with GD-NT and dimerizes upon AP20187 binding to achieve prolonged stability, effectively overcoming GD-CT’s low efficiency.

## Discussion

Gasdermins are predominantly expressed in the epithelium of the gastrointestinal tract and skin [39]. For a long time, gasdermins have been documented as regulators of epithelial cell growth, hair formation [40, 41], hearing loss [42], and carcinogenesis [43, 44]. In 2015, Gao et al. found that the GSDMA3 N-terminal fragment (GA3-NT) induces autophagy and subsequent cell death, which can be blocked by the GSDMA3 C-terminal fragment (GA3-CT) or the C-terminal fragments of other gasdermin members. The inhibitory function of GA3-CT was further emphasized by studying a loss-of-function mutant Y344H of full-length gasdma3 (GA3-FL-Y344H). Y344H, located in the C-terminal domain of GA3-FL, abolishes the inhibitory capability of the GA3 C-terminal domain and makes GA3-FL-Y344H toxic like GA3-NT [45]. Later, gasdermin-mediated cell death was identified as “pyroptosis” [5], which does not depend on autophagy. These findings suggest that gasdermins potentially regulate a variety of biological processes when being maintained in their full-length form [43, 44, 46], but specifically induce pyroptosis upon cleavage to release the cytolytic N-terminal fragments [47]. The intricacies of gasdermin processing remain to be explored for further understanding of the mechanisms underlying cell death.

GSDMD was the first characterized and is the most important pyroptosis executioner [5]. Structurally, GSDMD consists of a cytolytic *N*-terminal domain and an inhibitory C-terminal domain [48]. This intramolecular inhibitory mechanism renders GSDMD non-cytolytic and harmless to host cells [19]. To liberate the cytolytic activity of the *N*-terminal domain, inflammatory caspases cleave the linker region of GSDMD, separating it into two free fragments: GD-NT and GD-CT [5]. Although the pore-forming activity of GD-NT has been clarified [49], why GD-CT fails to efficiently block GD-NT-mediated pyroptosis remains poorly understood. In this study, we observed that GD-CT interacted with GD-NT and only partially blocked its cytolytic activity. Mechanistically, GD-NT translocates to and forms pores in the plasma membrane, whereas GD-CT is restricted to the cytoplasm. The spatial separation prevents effective inhibition. Additionally, GD-CT is an unstable protein with a short half-life in cells and is rapidly degraded via the proteasome-dependent pathway. Therefore, our results suggest that the low inhibitory efficiency of GD-CT results from its spatial separation and fast degradation.

Pyroptosis can be detrimental in conditions such as cancer, rheumatoid arthritis, inflammatory bowel disease, autoinflammatory syndromes, and neuroinflammation [15, 17, 50–52]. Given that GD-CT is not functionless but can regulate the extent of pyroptosis, it could potentially be bioengineered for therapeutic applications in pyroptosis-related diseases. To increase the inhibitory ability of GD-CT, we created the chimera protein FKBP-GD-CT by fusing GD-CT with an *N*-terminal Myr motif and two FKBP12^F36V^ domains. The Myr sequence targets the protein to the plasma membrane, bringing it to the site of GD-NT pore formation. The FKBP12^F36V^ domains, in the presence of the small molecule dimerizer AP20187, confer GD-CT a prolonged half-life. These two strategies significantly enhance the inhibitory efficiency of GD-CT.

In summary, we demonstrate the molecular basis underlying the limited inhibitory efficacy of GD-CT against GD-NT-mediated pyroptosis. To overcome this limitation, we engineered the chimera protein FKBP-GD-CT. Thus, our work helps to understand the regulation mechanisms of gasdermin-mediated pyroptosis and proposes innovative therapeutic strategies. Notably, our CID-based design of FKBP-GD-CT provides a uniquely powerful tool for dissecting pyroptosis mechanisms in vitro. Currently, in vivo delivery and tissue-specific expression of FKBP-GD-CT are technically challenging, hindering its therapeutic potential for pyroptosis-related diseases. Further studies are warranted to address these limitations.

## Methods

### Data reporting

No statistical methods were used to predetermine the sample size. The experiments were not randomized. Investigators were not blinded to allocation during experiments and outcome assessment.

### Cell lines and cell culture conditions

HEK293 cells (SCSP-502) and HeLa cells (SCSP-504) were obtained from the Cell Bank of the Chinese Academy of Sciences. Immortalized bone marrow-derived macrophages (iBMDM, EDC00071) were obtained from Editgene. These cell lines were maintained in Dulbecco’s Modified Eagle’s Medium (DMEM) supplemented with 10% heat-inactivated fetal bovine serum (FBS), 100 U/mL penicillin, and 100 µg/mL streptomycin at 37 °C under 5% CO₂. All cell lines were routinely screened for mycoplasma contamination using PCR and authenticated based on morphological characteristics.

### Plasmids

pDB-His-MBP-mGSDMD (Addgene, 123365) was a gift from Hao Wu and was used as a template to generate Flag-GD-CT, HA-GD-CT, Flag-GD-FL, Flag-GD-NT via the standard PCR cloning strategy. The cDNA sequences for nmFlag-GD-FL and FKBP-GD-CT constructs were generated through custom gene synthesis (Gencefe Biotech, China) and were subcloned into pcDNA3.1 or pTRIPZ-TetOn-based viral vectors via the standard PCR cloning strategy. An illustrative diagram of the structural composition of nmFlag-GD-FL and FKBP-GD-CT is shown in Fig. 4C and Fig. 6A, respectively. The amino acid sequences of all GSDMD constructs are provided in Extended Table 1. pcDNA3.1-based vectors were used for transient transfection. The pLenti-CMV-based viral vector and pTRIPZ-TetOn-based viral vector were used to create 293-tetO-GD-NT cells and iBMDM-cFKBP-GD-CT cells, respectively. All plasmids were verified by DNA sequencing and immunoblot analysis.

### Reagents and antibodies

Doxycycline (D3447) was obtained from Sigma-Aldrich. Subcellular Protein Fractionation Kit (78840) was obtained from Thermo Fisher. StrataClean resin (400714) and chemical competent cells (200315) were obtained from Agilent. For immunoblotting, anti-Flag (F1804), anti-Flag (F7425), and anti-HA (H6908) were obtained from Sigma-Aldrich. Anti-GSDMD (ab219800) was obtained from Abcam. pS166-RIP (65746), pS227-RIP3 (93654), pS358-MLKL (91689), LC3B (83506) and p62 (88588) were obtained from CST. Anti-β-Actin (sc-47778), anti-GST (sc-138), and anti-Na/K-ATPase α1 (sc-21712) were purchased from Santa Cruz Biotechnology. For immunofluorescence, anti-Rabbit conjugated with FITC (GB22303) and anti-Mouse conjugated with AF594 (GB28303) were obtained from Servicebio.

### Stable cell lines

Lentivirus was produced in HEK293 cells by transfection of the lentiviral vector with psPAX2 (Addgene) and pMD2.G (Addgene). Lentiviral supernatants were collected, filtered through 0.45 µm filters, and used to transduce HEK293 cells. Polybrene infection/transfection reagent (Millipore, 10 µg/mL) was added to increase the efficiency of lentiviral infection. After 2 days of transduction, puromycin (Sigma, 2 µg/mL) was added to select the transduced cells. Empty lentiviral vectors were used to generate control cells. The expression of relevant genes in stable cell lines was verified by immunoblot.

### Cytotoxicity assay and cell viability assay

To quantify pyroptotic cell death, cell death and cell viability were performed using the Non-Radioactive Cytotoxicity Assay kit (G1780, Promega) and the CellTiter-Glo Luminescent Cell Viability Assay kit (G7571, Promega), respectively, according to the manufacturer’s instructions. Briefly, 5 × 10^3^ cells were cultured in 96-well plates with opaque walls. At the desired time points, cell death was determined by titrating the amount of lactate dehydrogenase (LDH) released into the culture medium. The maximum LDH control was obtained by lysing cells with the kit-provided LDH Release Reagent. Absorbance was measured at 490 nm using a multifunctional microplate reader (PerkinElmer EnSight). Cell viability was determined based on the ATP levels within cells, and the corresponding luminescence signals were quantified using the same PerkinElmer EnSight platform.

### Immunoblots and immunoprecipitation

Cells were lysed in EBC buffer (50 mM Tris pH 7.5, 120 mM NaCl, 0.5% NP-40) supplemented with protease inhibitors (A32953, Thermo Fisher) and phosphatase inhibitors (B15002, Bimake). Protein concentrations of cell lysates were measured using the Beckman Coulter DU-800 spectrophotometer and the Bio-Rad protein assay reagent. Equal amounts of total proteins were resolved by SDS-PAGE and immunoblotted with the indicated antibodies. For immunoprecipitation, cell lysates containing 1 mg of total proteins were incubated with anti-Flag agarose (A2220, Sigma) or anti-HA agarose (A2095, Sigma) for 4 hours at 4 °C. Precipitates were washed three times with EBC buffer and resolved by SDS-PAGE, followed by immunoblot analysis with the indicated antibodies.

### Pyroptosis detection by flow cytometry

Cell pyroptosis was quantified using an Annexin V-FITC/PI detection kit (KeyGEN BioTECH, KGA1102) according to manufacturer guidelines. Briefly, 1–5 × 10⁵ cells were collected using EDTA-free trypsin with strict limitation of digestion time to minimize false positives. After PBS washes (300 × g, 5 min), the cells were resuspended in 500 μL binding Buffer. Dual staining was performed by adding 5 μL Annexin V-FITC and 5 μL propidium iodide (PI), followed by gentle mixing and incubation at room temperature for 15 min, protected from light. Samples were acquired using the BD Accuri™ C6 Flow Cytometer (BD Biosciences) and analyzed using FlowJo software (Tree Star).

### Immunofluorescence staining and microscopy

For immunofluorescence staining, the dishes with a special glass bottom were used. Cells were fixed with 100% methanol (chilled at −20 °C) at room temperature for 5 min. To minimize nonspecific binding of the antibodies, the cells were incubated with blocking solution containing 1% BSA, 22.52 mg/mL glycine in PBST (PBS + 0.1% Tween 20) for 30 min. For immunostaining, cells were incubated with the primary antibody in blocking solution (1% BSA in PBST) overnight at 4 °C in a humidified chamber and subsequently incubated with the secondary antibody in blocking solution for 1 h at room temperature in the dark. The cells were counterstained with DAPI (1 µg/mL), mounted, and subjected to imaging under the LSM 880 confocal microscope (Carl Zeiss AG, Germany) or the Axio Observer 3 widefield fluorescence microscope (Carl Zeiss AG, Germany).

For fluorescence microscopy of living cells, PI (10 µg/mL) or DAPI (1 µg/mL) was added to the culture medium. 10 minutes later, the cells were imaged under the same widefield fluorescence microscope.

### Statistical analysis

Student’s t-test was used to determine the differences between the two groups. The results are presented as the mean and standard error of the mean (s.e.m). Statistical significance was assigned to P < 0.05.

## Supplementary information

Uncropped original western blot

Supplemental Material

## Data Availability

Original data are available upon request. The full length uncropped original western blots are shown in the ‘Supplementary Material’.
