# Supplementary material for: Chemically induced dimerization of GSDMD C-terminal domain blocks GSDMD N-terminal domain-mediated pyroptosis
Source: Cell Death Discov. 2025 Oct 13;11:456. doi: 10.1038/s41420-025-02733-0 (PMC12518871; doi:10.1038/s41420-025-02733-0)
Supplement: Supplementary file 1 — Uncropped original western blot [file 41420_2025_2733_MOESM1_ESM.pptx]

## Slide 1
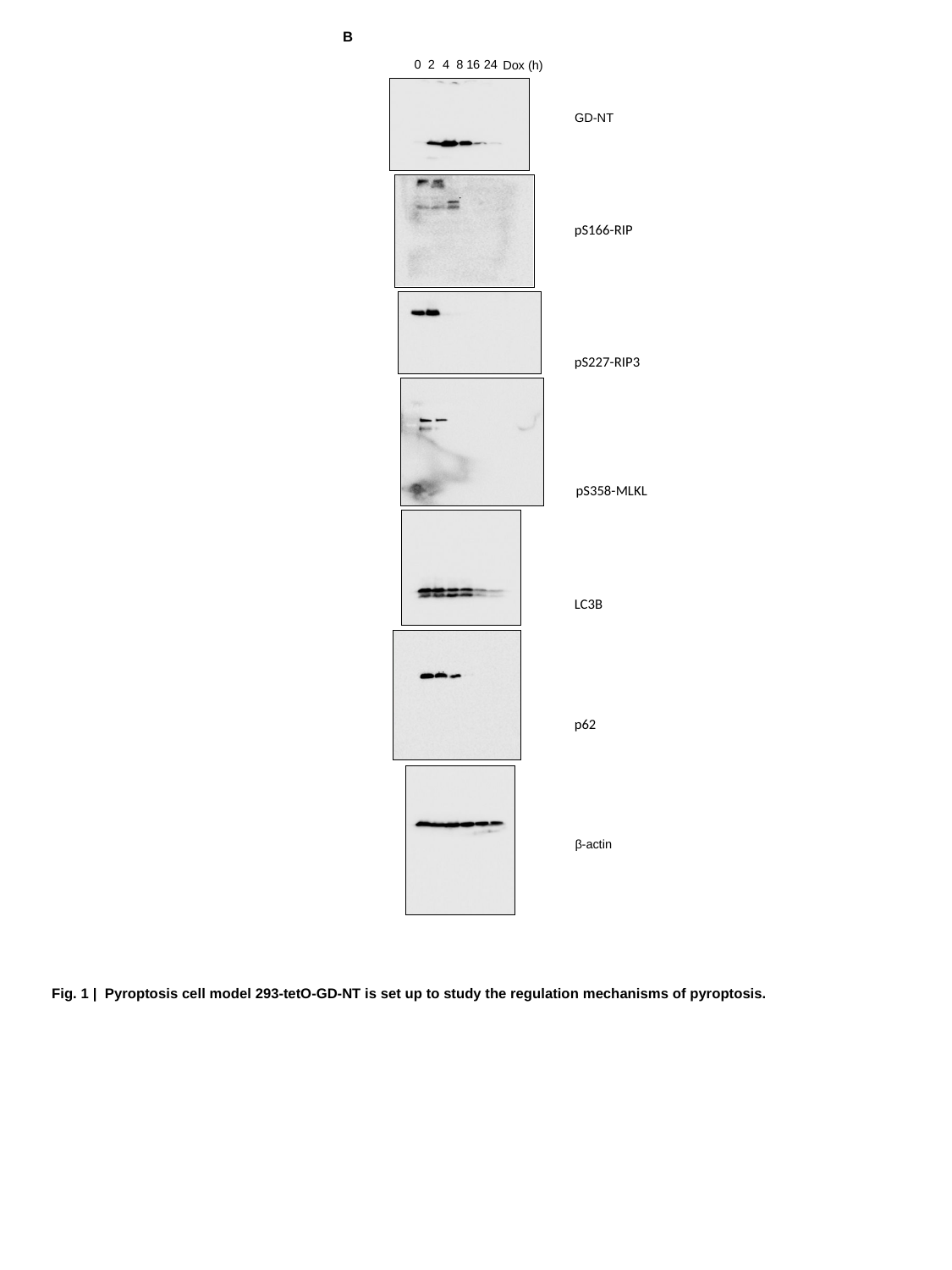

B
0
2
4
8
16
24
Dox (h)
GD-NT
pS166-RIP
pS227-RIP3
pS358-MLKL
LC3B
p62
β-actin
Fig. 1 | Pyroptosis cell model 293-tetO-GD-NT is set up to study the regulation mechanisms of pyroptosis.

## Slide 2
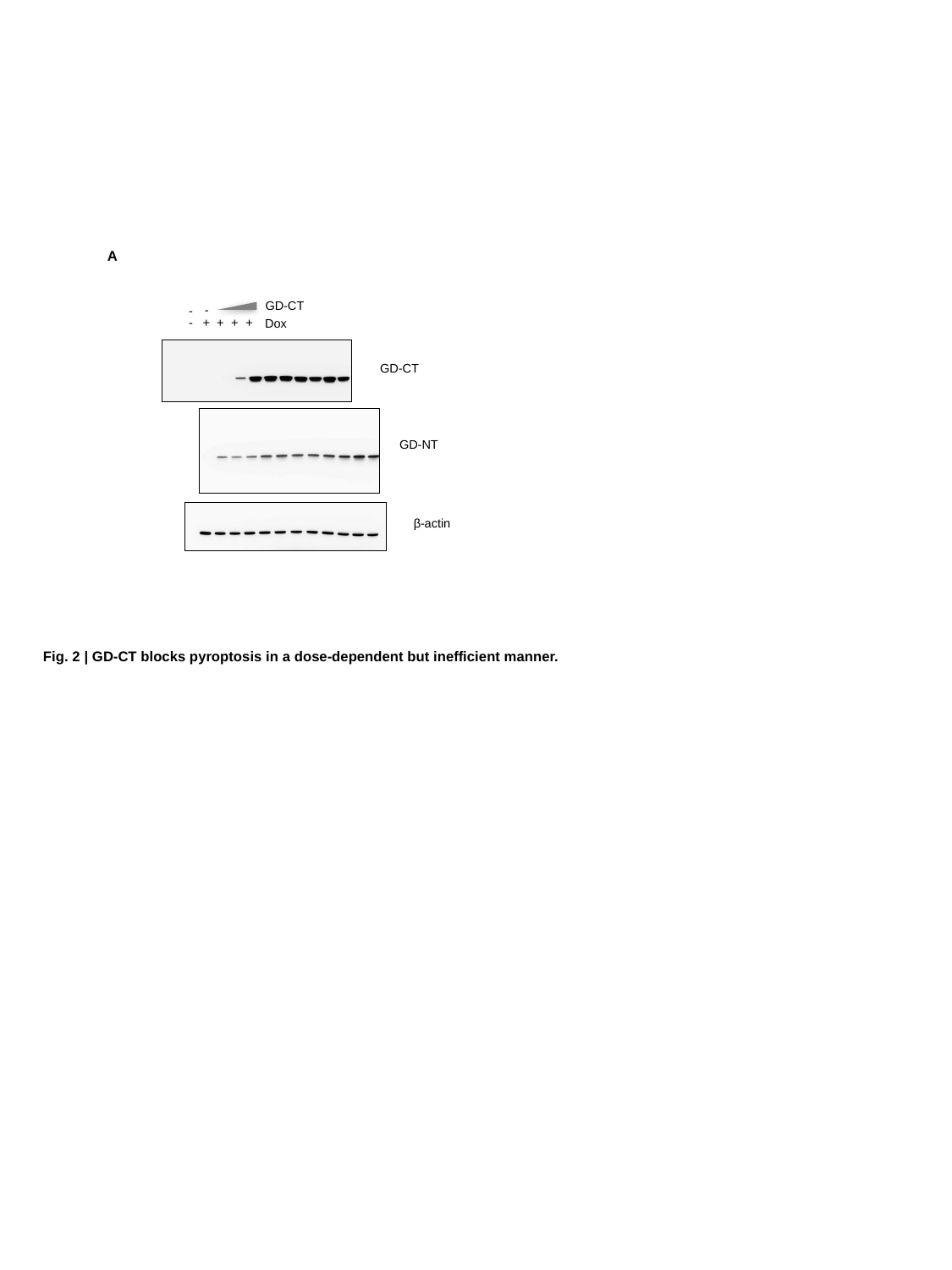

A
-
-
GD-CT
+
+
+
+
-
Dox
GD-CT
GD-NT
β-actin
Fig. 2 | GD-CT blocks pyroptosis in a dose-dependent but inefficient manner.

## Slide 3
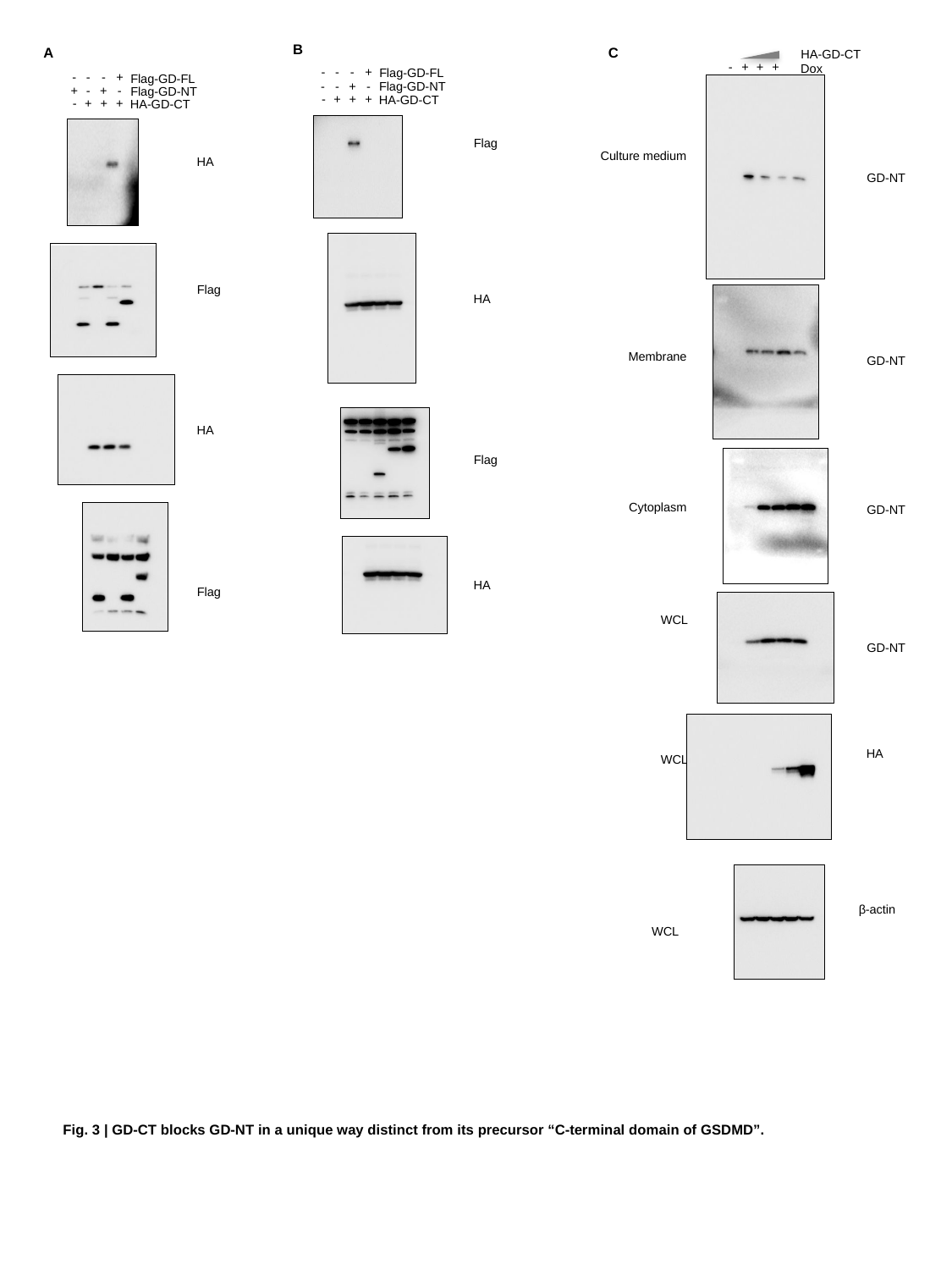

B
C
A
HA-GD-CT
-
+
+
+
Dox
-
-
-
+
Flag-GD-FL
-
-
-
+
Flag-GD-FL
-
-
+
-
Flag-GD-NT
+
-
+
-
Flag-GD-NT
-
+
+
+
HA-GD-CT
-
+
+
+
HA-GD-CT
Flag
Culture medium
HA
GD-NT
Flag
HA
Membrane
GD-NT
HA
Flag
Cytoplasm
GD-NT
HA
Flag
WCL
GD-NT
HA
WCL
β-actin
WCL
Fig. 3 | GD-CT blocks GD-NT in a unique way distinct from its precursor “C-terminal domain of GSDMD”.

## Slide 4
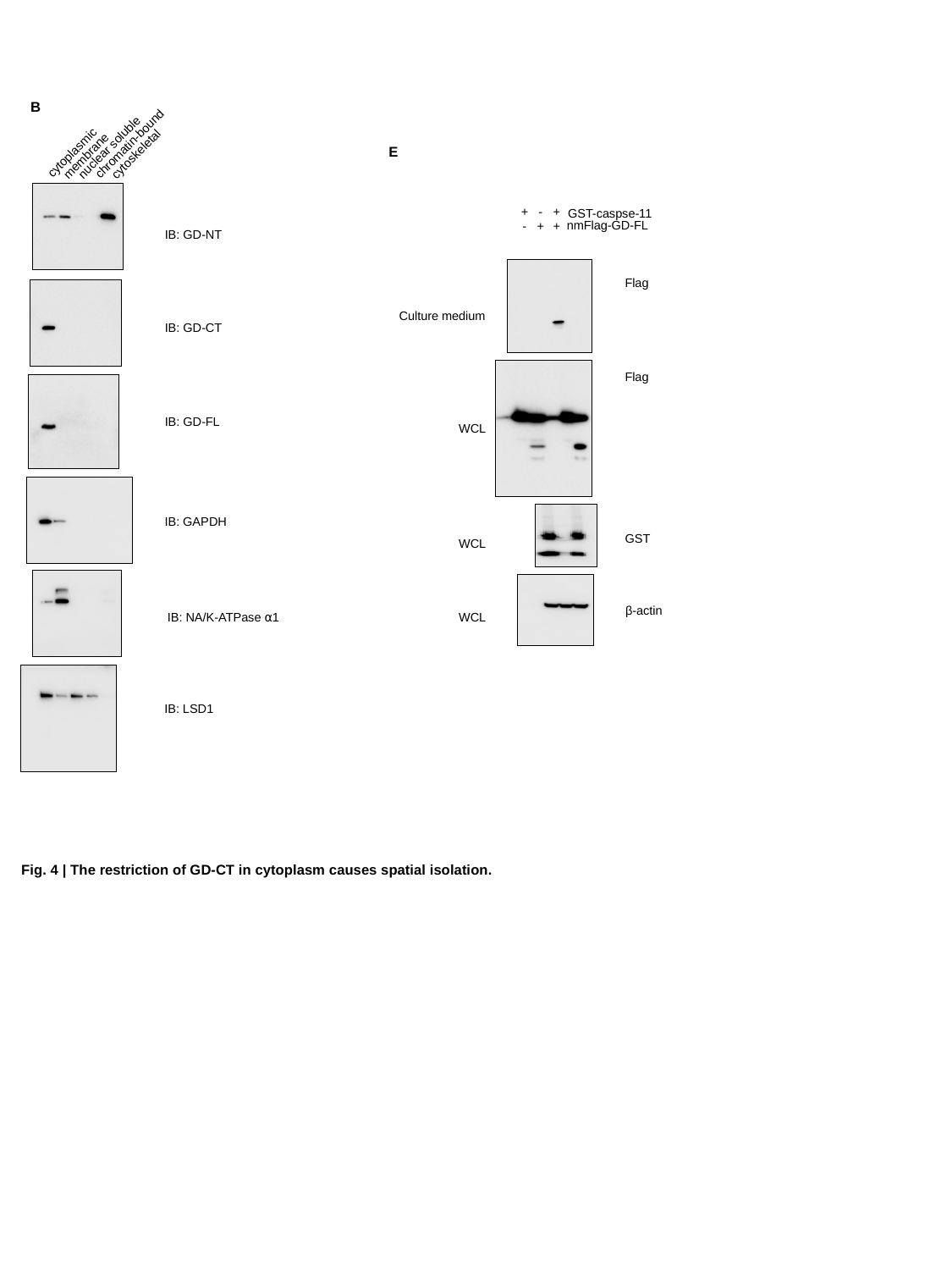

B
chromatin-bound
nuclear soluble
E
cytoplasmic
cytoskeletal
membrane
+
-
+
GST-caspse-11
nmFlag-GD-FL
-
+
+
IB: GD-NT
Flag
Culture medium
IB: GD-CT
Flag
IB: GD-FL
WCL
IB: GAPDH
GST
WCL
β-actin
IB: NA/K-ATPase ⍺1
WCL
IB: LSD1
Fig. 4 | The restriction of GD-CT in cytoplasm causes spatial isolation.

## Slide 5
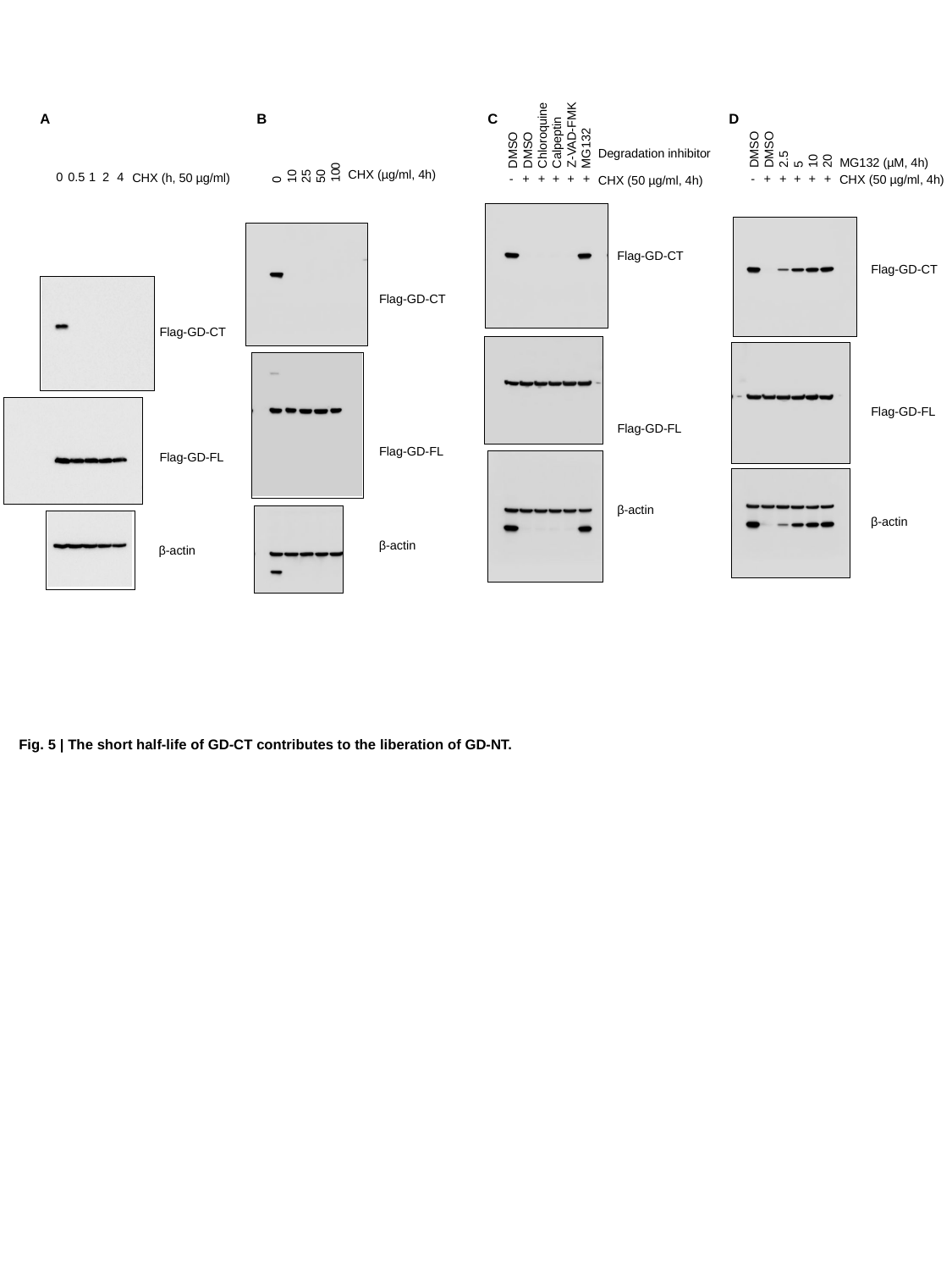

A
B
C
D
Z-VAD-FMK
Chloroquine
Calpeptin
DMSO
DMSO
DMSO
DMSO
MG132
Degradation inhibitor
2.5
10
MG132 (µM, 4h)
20
5
100
CHX (µg/ml, 4h)
10
25
50
0
0.5
1
2
4
CHX (h, 50 µg/ml)
-
+
+
+
+
+
-
+
+
+
+
+
0
CHX (50 µg/ml, 4h)
CHX (50 µg/ml, 4h)
Flag-GD-CT
Flag-GD-CT
Flag-GD-CT
Flag-GD-CT
Flag-GD-FL
Flag-GD-FL
Flag-GD-FL
Flag-GD-FL
β-actin
β-actin
β-actin
β-actin
Fig. 5 | The short half-life of GD-CT contributes to the liberation of GD-NT.

## Slide 6
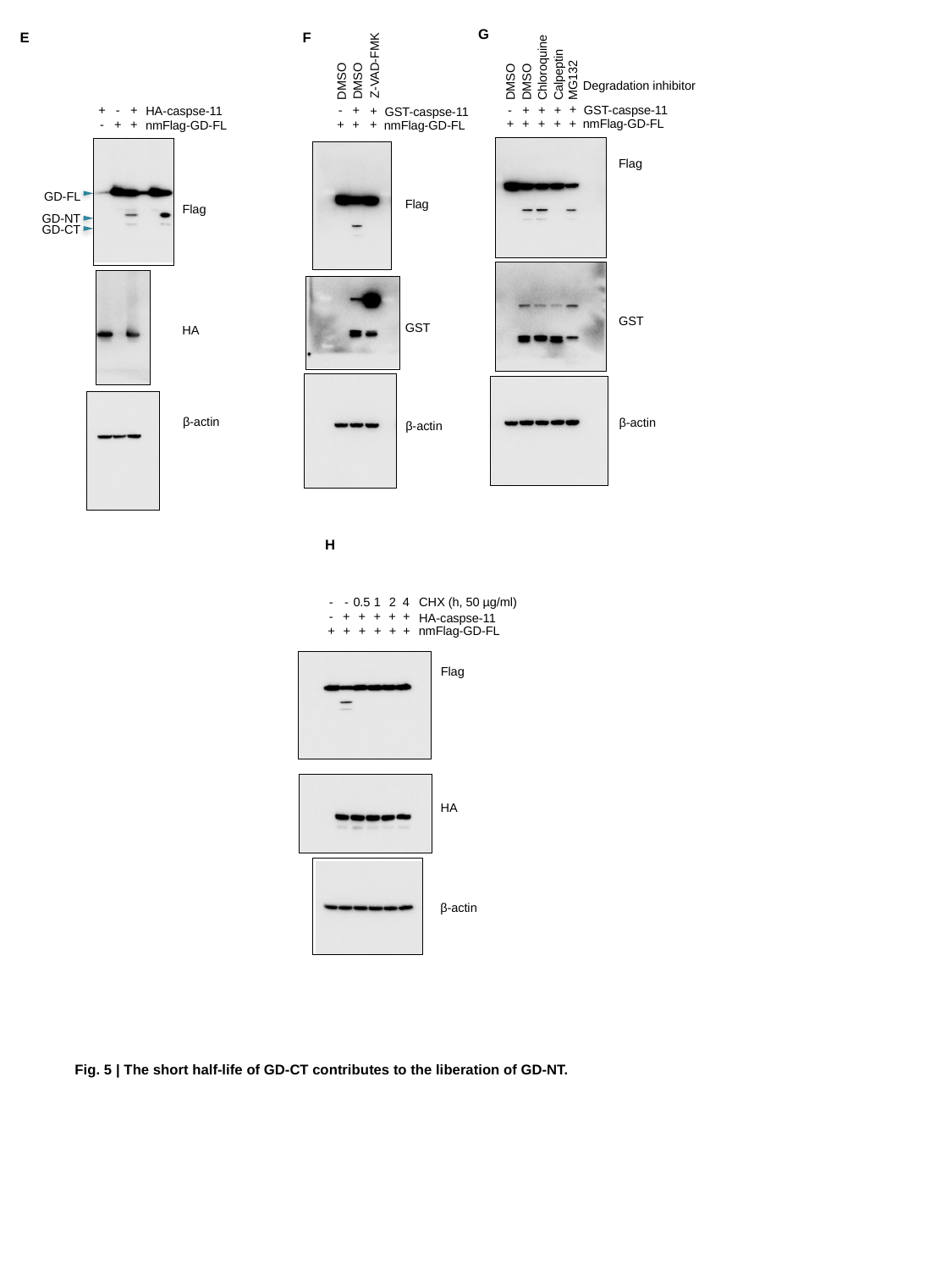

G
E
F
Z-VAD-FMK
Chloroquine
Calpeptin
MG132
DMSO
DMSO
DMSO
DMSO
Degradation inhibitor
+
GST-caspse-11
+
+
+
-
+
+
-
+
-
+
HA-caspse-11
GST-caspse-11
+
+
+
+
+
nmFlag-GD-FL
+
+
+
-
+
+
nmFlag-GD-FL
nmFlag-GD-FL
Flag
GD-FL
Flag
Flag
GD-NT
GD-CT
GST
GST
HA
β-actin
β-actin
β-actin
H
-
-
CHX (h, 50 µg/ml)
0.5
1
2
4
-
+
+
+
+
+
HA-caspse-11
+
+
+
+
+
+
nmFlag-GD-FL
Flag
HA
β-actin
Fig. 5 | The short half-life of GD-CT contributes to the liberation of GD-NT.

## Slide 7
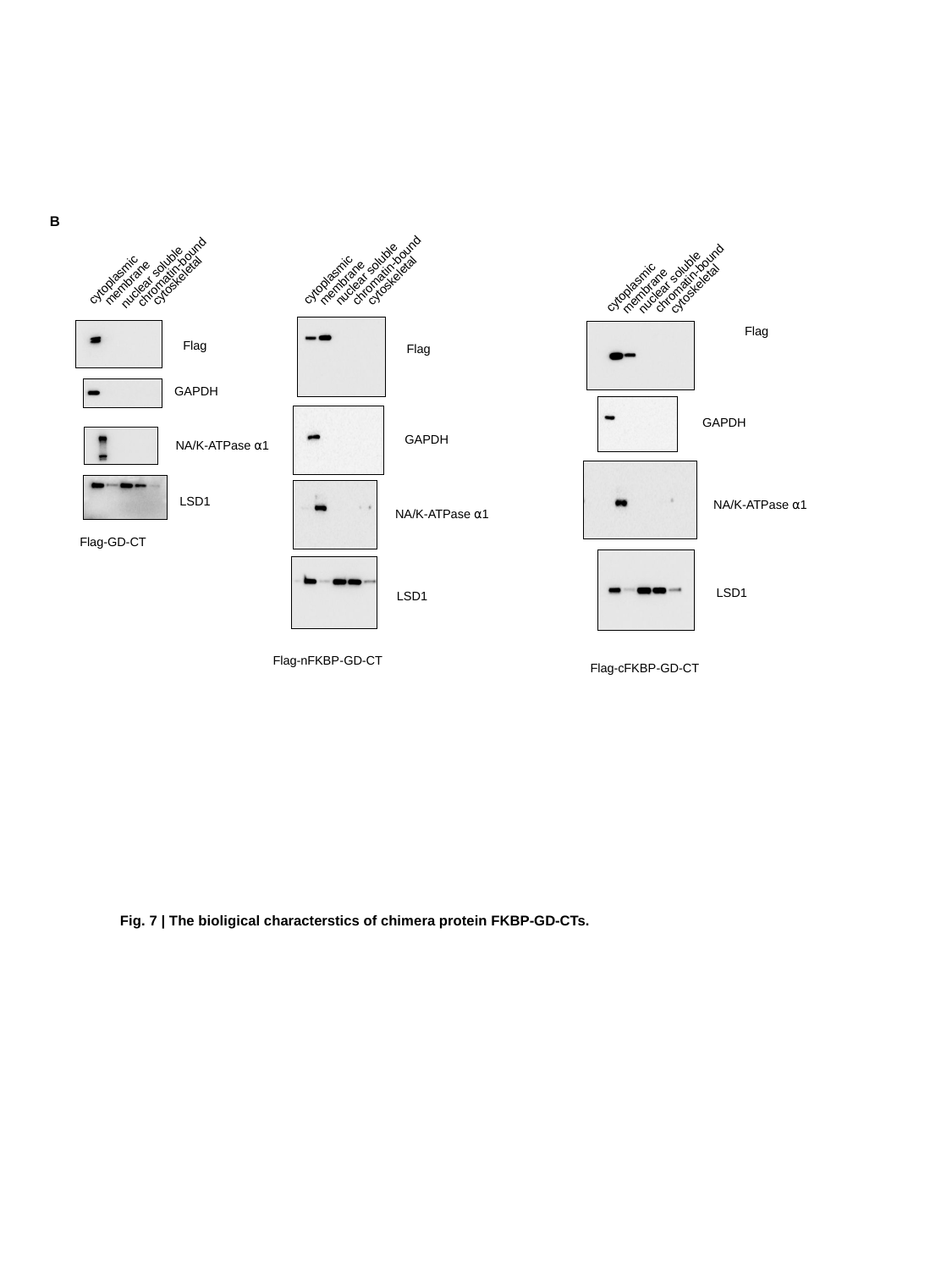

B
chromatin-bound
chromatin-bound
nuclear soluble
nuclear soluble
chromatin-bound
cytoplasmic
cytoplasmic
cytoskeletal
cytoskeletal
nuclear soluble
membrane
membrane
cytoplasmic
cytoskeletal
membrane
Flag
Flag
Flag
GAPDH
GAPDH
GAPDH
NA/K-ATPase ⍺1
LSD1
NA/K-ATPase ⍺1
NA/K-ATPase ⍺1
Flag-GD-CT
LSD1
LSD1
Flag-nFKBP-GD-CT
Flag-cFKBP-GD-CT
Fig. 7 | The bioligical characterstics of chimera protein FKBP-GD-CTs.

## Slide 8
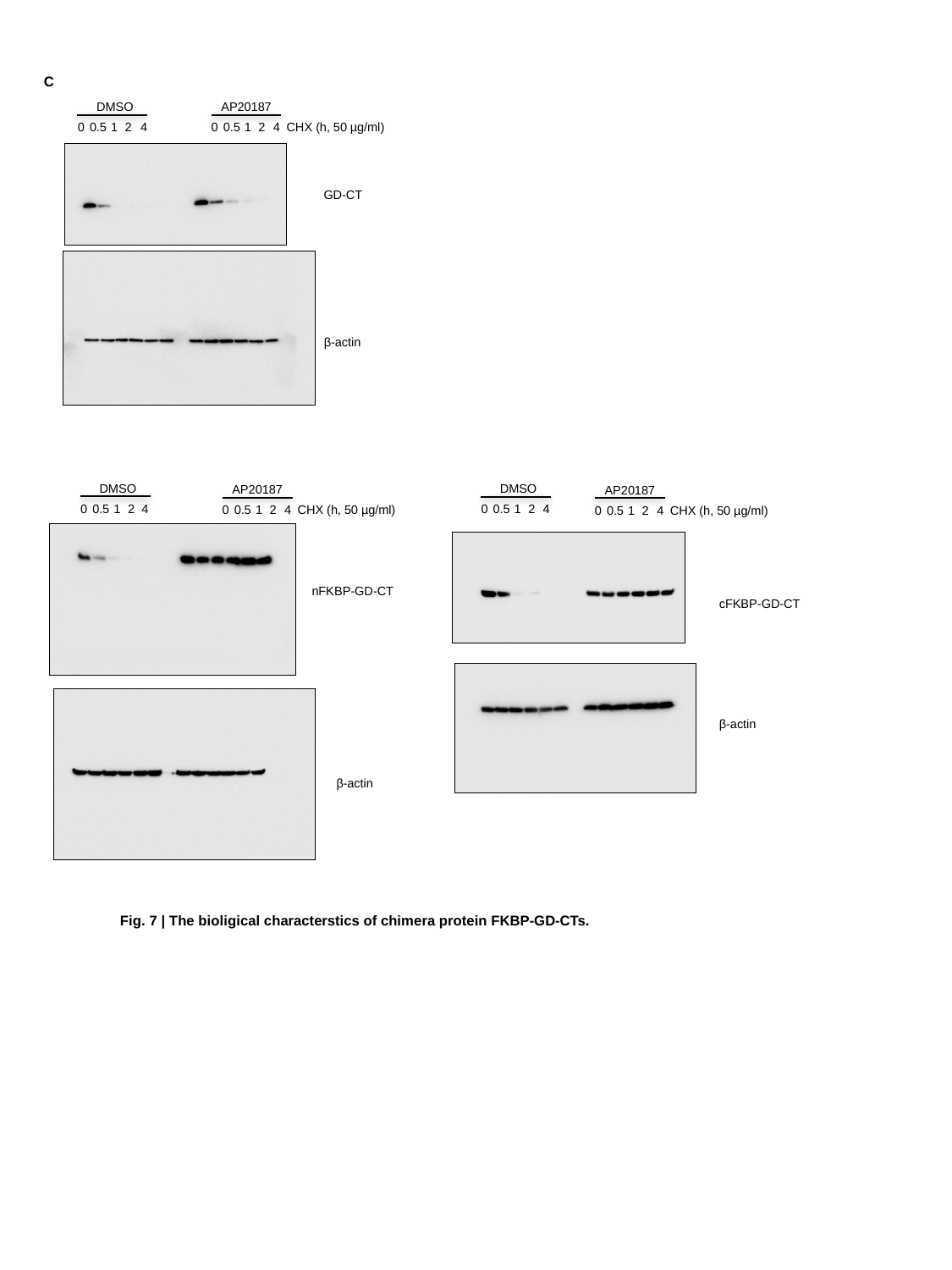

C
DMSO
AP20187
0
0.5
1
2
4
0
0.5
1
2
4
CHX (h, 50 µg/ml)
GD-CT
β-actin
DMSO
DMSO
AP20187
AP20187
4
0
0.5
1
2
0
0.5
1
2
4
0
0.5
1
2
4
CHX (h, 50 µg/ml)
0
0.5
1
2
4
CHX (h, 50 µg/ml)
nFKBP-GD-CT
cFKBP-GD-CT
β-actin
β-actin
Fig. 7 | The bioligical characterstics of chimera protein FKBP-GD-CTs.

## Slide 9
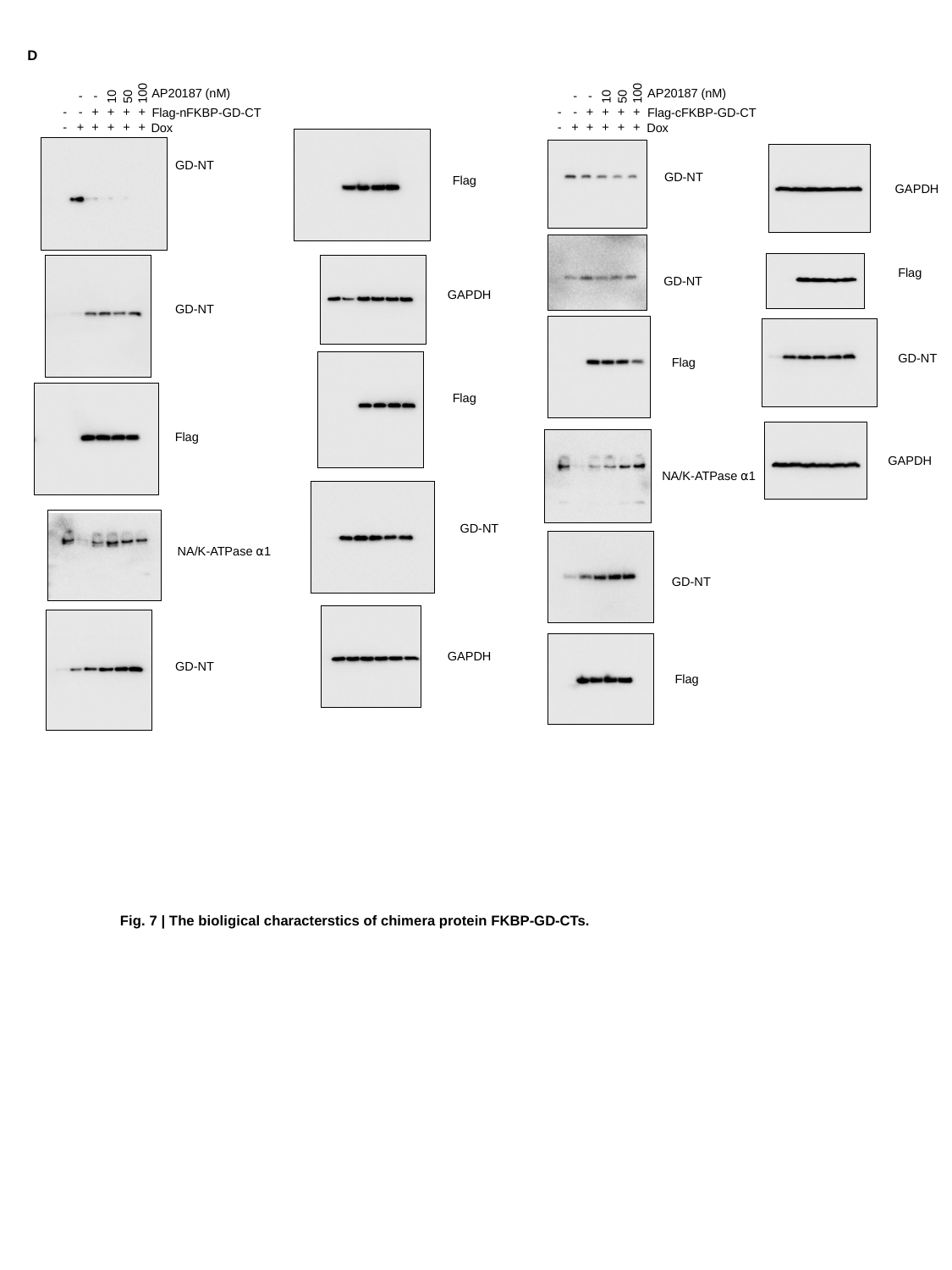

D
100
100
AP20187 (nM)
AP20187 (nM)
-
-
-
-
10
50
10
50
-
-
+
+
+
+
-
-
+
+
+
+
Flag-nFKBP-GD-CT
Flag-cFKBP-GD-CT
-
+
+
+
+
+
-
+
+
+
+
+
Dox
Dox
GD-NT
GD-NT
Flag
GAPDH
Flag
GD-NT
GAPDH
GD-NT
GD-NT
Flag
Flag
Flag
GAPDH
NA/K-ATPase ⍺1
GD-NT
NA/K-ATPase ⍺1
GD-NT
GAPDH
GD-NT
Flag
Fig. 7 | The bioligical characterstics of chimera protein FKBP-GD-CTs.

## Slide 10
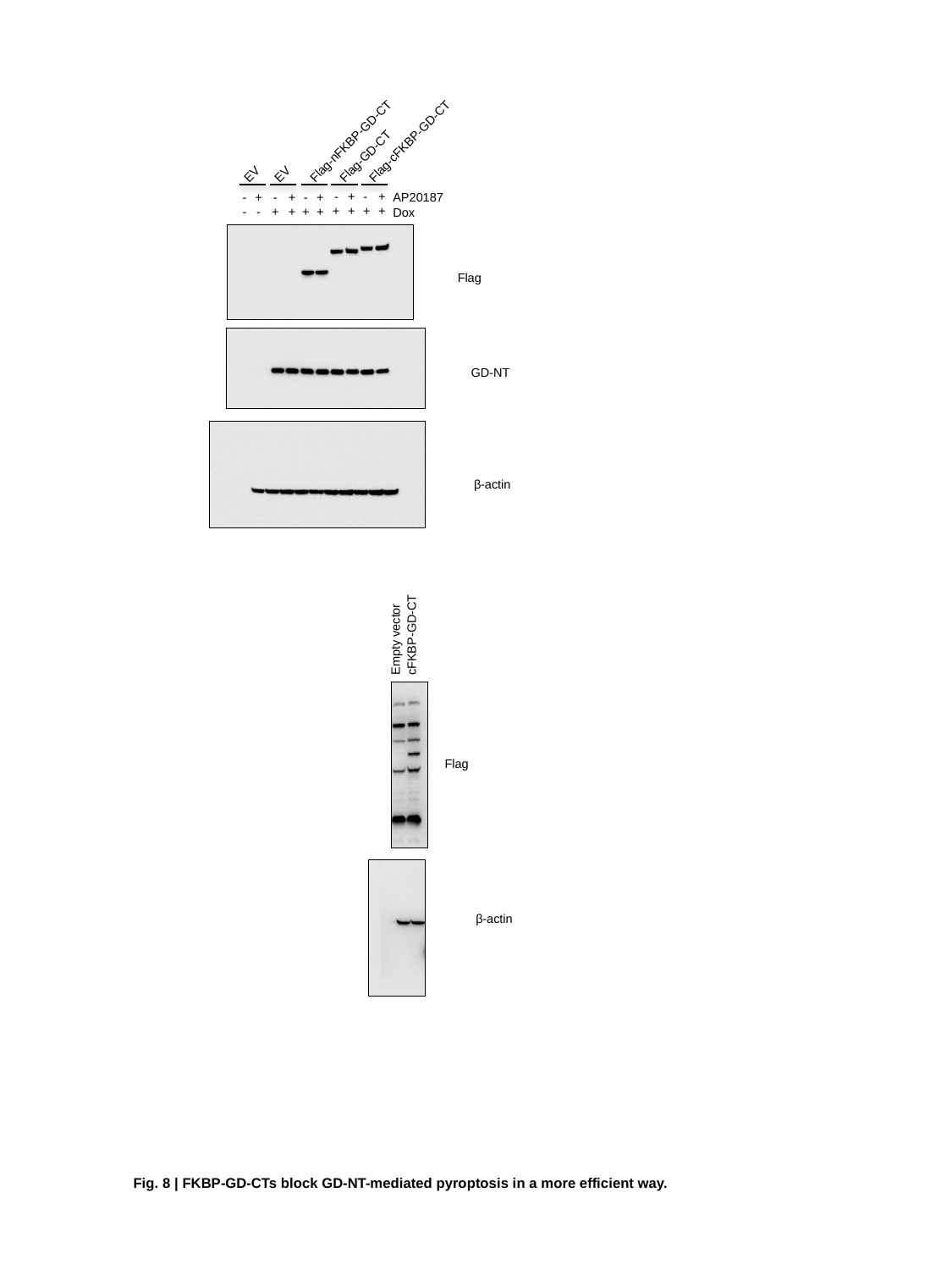

Flag-nFKBP-GD-CT
Flag-cFKBP-GD-CT
Flag-GD-CT
EV
EV
-
+
-
+
AP20187
-
+
-
+
-
+
+
+
+
+
-
-
+
+
+
+
Dox
Flag
GD-NT
β-actin
cFKBP-GD-CT
Empty vector
Flag
β-actin
Fig. 8 | FKBP-GD-CTs block GD-NT-mediated pyroptosis in a more efficient way.

## Slide 11
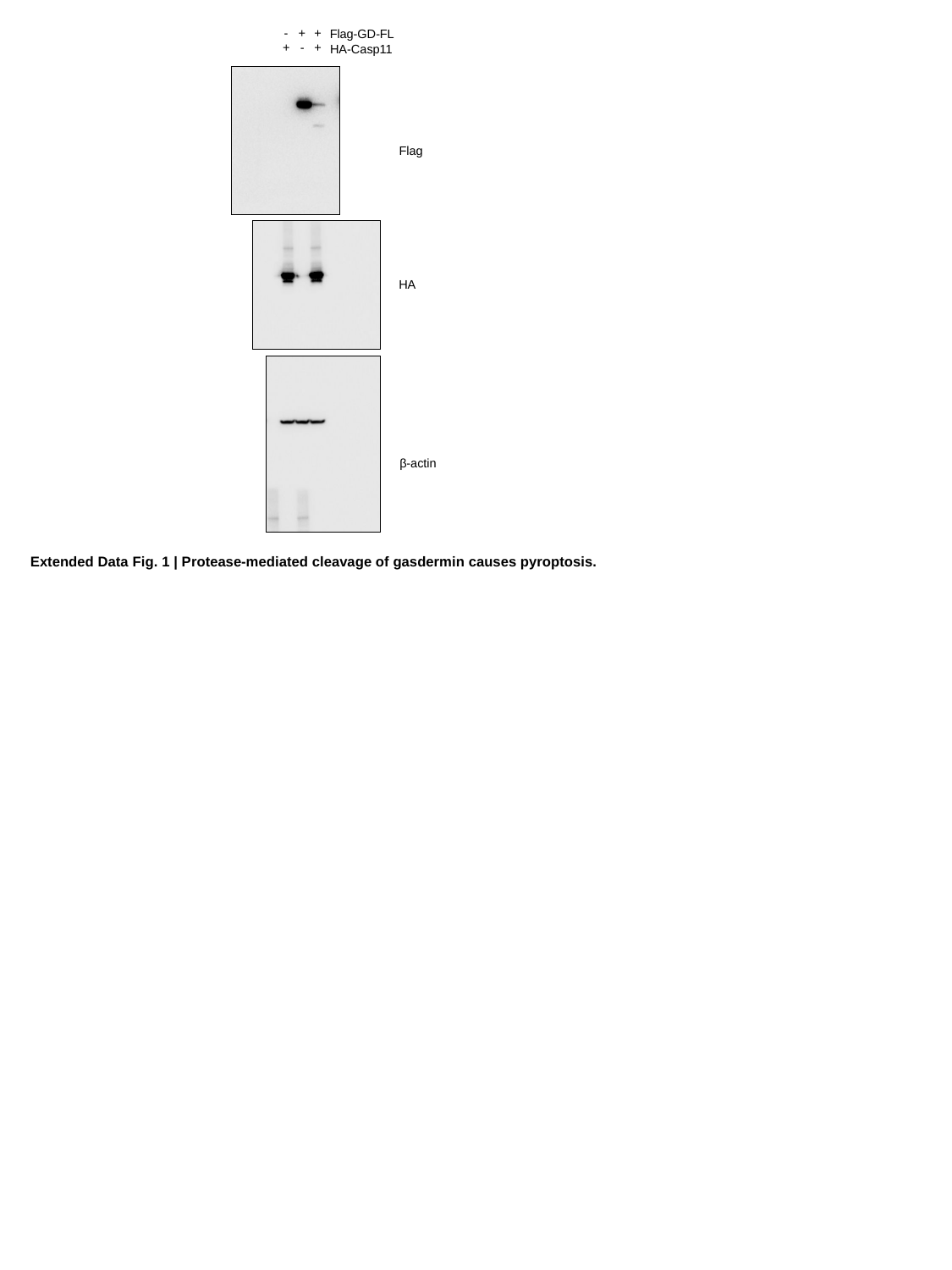

+
+
-
Flag-GD-FL
-
+
+
HA-Casp11
Flag
HA
β-actin
Extended Data Fig. 1 | Protease-mediated cleavage of gasdermin causes pyroptosis.
