# Supplementary material for: Chemically induced dimerization of GSDMD C-terminal domain blocks GSDMD N-terminal domain-mediated pyroptosis
Source: Cell Death Discov. 2025 Oct 13;11:456. doi: 10.1038/s41420-025-02733-0 (PMC12518871; doi:10.1038/s41420-025-02733-0)
Supplement: Supplementary file 2 — Supplemental Material [file 41420_2025_2733_MOESM2_ESM.pdf]

Supplementary Materials for

Chemically induced dimerization of GSDMD C-terminal domain blocks GSDMD N-terminal domain-mediated pyroptosis

Jixuan Xu[1, 2], Miaoran Fu[3], Yamin Xing[4], Wulong Liang[5], Guangyuan Li[2], Ting Zhang[2], Mengxue Li[4], Chunxiao Gao[2], Zhanfeng Yang[1], Yuming Fu[1], Min Zhang[3], Zisen Zhang[2, 6], Pengyuan Zheng[4], Xiufeng Chu[2, 4, 6]*

[1]Department of Gastrointestinal & Thyroid Surgery, The Fifth Affiliated Hospital of Zhengzhou University, Zhengzhou, China.

[2]Department of Oncology, The Fifth Affiliated Hospital of Zhengzhou University, Zhengzhou, China.

[3]Department of Neurology, The Fifth Affiliated Hospital of Zhengzhou University, Zhengzhou, China.

[4]Marshall B. J. Medical Center, The Fifth Affiliated Hospital of Zhengzhou University, Zhengzhou, China.

[5]Henan International Joint Laboratory of Glioma Metabolism and Microenvironment Research, The Fifth Affiliated Hospital of Zhengzhou University, Zhengzhou University, Zhengzhou, China

[6]Tianjian Laboratory of Advanced Biomedical Sciences, Zhengzhou 450000, Henan, China.

* Corresponding authors:

Xiufeng Chu: chuxiufeng831031@gmail.com

This file includes:

Extended Fig. 1-2 and Extended Table 1

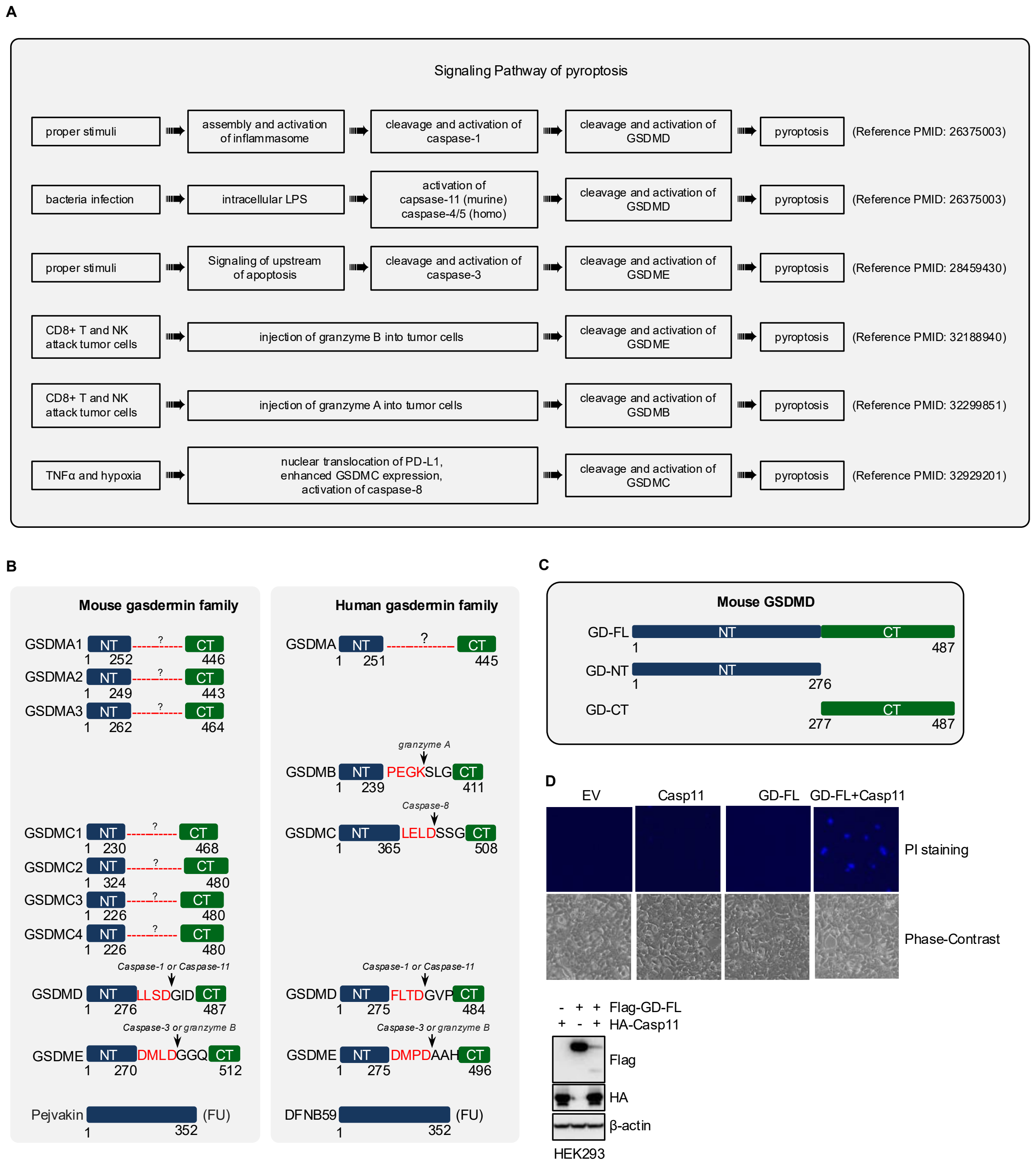
A
Signaling Pathway of pyroptosis
proper stimuli → assembly and activation of inflammasome → cleavage and activation of caspase-1 → cleavage and activation of GSDMD → pyroptosis (Reference PMID: 26375003)
bacteria infection → intracellular LPS → activation of capsase-11 (murine) caspase-4/5 (homo) → cleavage and activation of GSDMD → pyroptosis (Reference PMID: 26375003)
proper stimuli → Signaling of upstream of apoptosis → cleavage and activation of caspase-3 → cleavage and activation of GSDME → pyroptosis (Reference PMID: 28459430)
CD8+ T and NK attack tumor cells → injection of granzyme B into tumor cells → cleavage and activation of GSDME → pyroptosis (Reference PMID: 32188940)
CD8+ T and NK attack tumor cells → injection of granzyme A into tumor cells → cleavage and activation of GSDMB → pyroptosis (Reference PMID: 32299851)
TNFα and hypoxia → nuclear translocation of PD-L1, enhanced GSDMC expression, activation of caspase-8 → cleavage and activation of GSDMC → pyroptosis (Reference PMID: 32929201)
B
Mouse gasdermin family
GSDMA1 NT ? CT 1 252 446
GSDMA2 NT ? CT 1 249 443
GSDMA3 NT ? CT 1 262 464
GSDMC1 NT ? CT 1 230 468
GSDMC2 NT ? CT 1 324 480
GSDMC3 NT ? CT 1 226 480
GSDMC4 NT ? CT 1 226 480
Caspase-1 or Caspase-11
GSDMD NT LLSDGID CT 1 276 487
Caspase-3 or granzyme B
GSDME NT DMLDGGQ CT 1 270 512
Pejvakin (FU) 1 352
Human gasdermin family
GSDMA NT ? CT 1 251 445
granzyme A
GSDMB NT PEGKSLG CT 1 239 411
Caspase-8
GSDMC NT LELDSSG CT 1 365 508
Caspase-1 or Caspase-11
GSDMD NT FLTDGVP CT 1 275 484
Caspase-3 or granzyme B
GSDME NT DMPDAAH CT 1 275 496
DFNB59 (FU) 1 352
C
Mouse GSDMD
GD-FL NT CT 1 487
GD-NT NT 1 276
GD-CT CT 277 487
D
EV
Casp11
GD-FL
GD-FL+Casp11
PI staining
Phase-Contrast
- + + Flag-GD-FL
+ - + HA-Casp11
Flag
HA
β-actin
HEK293

Extended Fig. 1 | Protease-mediated cleavage of gasdermin causes pyroptosis.
A. Schematic illustration of pyroptotic signaling pathways indicates that the specific cleavage and activation of gasdermin family is the key event of pyroptosis.
B. Schematic illustration of the cleavage site in human or mouse gasdermin family and the proteases responsible for the specific cleavage.
C. Schematic illustration of mouse GSDMD full-length (GD-FL), N-terminal domain (GD-NT) and C-terminal domain (GD-CT).
D. Caspase-11 cleaves GSDMD to produce the cytolytic fragment GD-NT. HEK293 cells were transfected with Flag-GD-FL and/or HA-caspase-11. 24 hour later, cells were subjected to DAPI staining for fluorescent microscope imaging or cell lysis for immunoblot with the indicated antibodies.

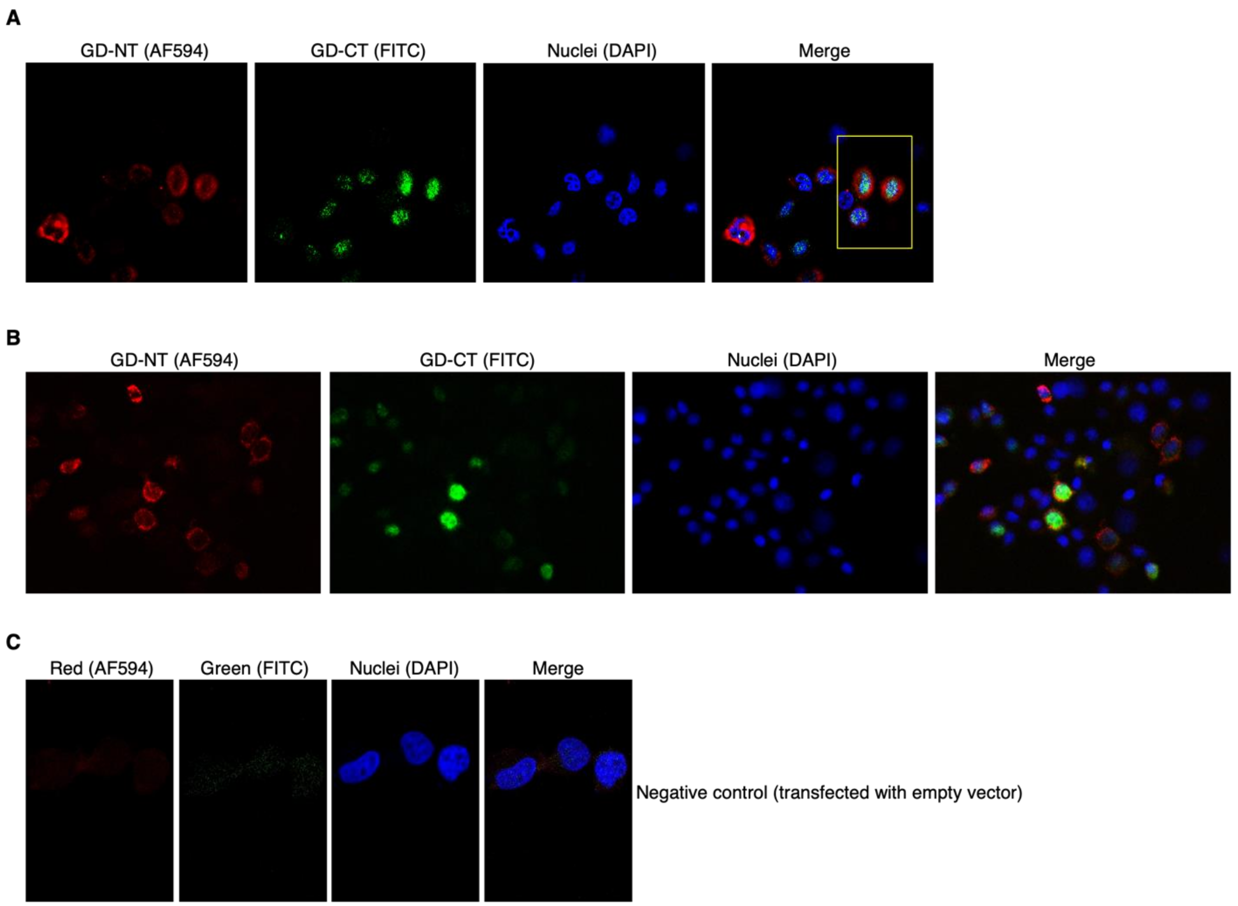

Extended Data Fig. 2 | The restriction of GD-CT in cytoplasm causes a spatial isolation.
A. Uncropped immunofluorescence images corresponding to Fig. 4A. Yellow box indicates the region shown in the main figure.
B. Similar to (A), except that subcellular localization of GD-NT and GD-CT was analyzed using a widefield fluorescence microscope.
C. Antibody specificity validation. To assess the specificity of fluorescent-conjugated antibodies used in the immunofluorescence assay, HeLa cells transfected with empty

vector (negative control) were subjected to identical fixation and staining procedures as in experiments (A) and (B). Non-specific staining was analyzed by confocal microscopy. Absence of detectable signal in negative control confirmed antibody specificity.

Extended Table 1 | Amino acid sequence of GSDMD constructs

GD-FL	MPSAFEKVVKNVIKEVSGSRGDLIPVDSLRNSTSFRPYCLLNRKFSSSRF<br>WKPRYSCVNLSIKDILEPSAPEPEPECFGSFKVSDVVDGNIQGRVMLSGM<br>GEGKISGGAAVSDSSSASMNVCILRVTQKTWETMQHERHLQQPENKILQ<br>QLRSRGDDLFVVTEVLQTKEEVQITEVHSQEGSGQFTLPGALCLKGEGKG<br>HQSRKKMVTIPAGSILAFRVAQLLIGSKWDILLVSDEKQRTFEPSSGDRKAV<br>GQRHHGLNVLAALCSIGKQLSLLSDGIDEEELIEAADFQGLYAEVKACSSE<br>LESLEMELRQQILVNIGKILQDQPSMEALEASLGQGLCSGGQVEPLDGPA<br>GCILECLVLDSGELVPELAAPIFYLLGALAVLSETQQQLLAKALETTVLSKQL<br>ELVKHVLEQSTPWQEQSSVSLPTVLLGDCWDEKNPTWVLLEECGLRLQV<br>ESPQVHWEPTSLIPTSALYASLFLLSSLGQKPC
GD-NT	MPSAFEKVVKNVIKEVSGSRGDLIPVDSLRNSTSFRPYCLLNRKFSSSRF<br>WKPRYSCVNLSIKDILEPSAPEPEPECFGSFKVSDVVDGNIQGRVMLSGM<br>GEGKISGGAAVSDSSSASMNVCILRVTQKTWETMQHERHLQQPENKILQ<br>QLRSRGDDLFVVTEVLQTKEEVQITEVHSQEGSGQFTLPGALCLKGEGKG<br>HQSRKKMVTIPAGSILAFRVAQLLIGSKWDILLVSDEKQRTFEPSSGDRKAV<br>GQRHHGLNVLAALCSIGKQLSLLSD
GD-CT	MGIDEEELIEAADFQGLYAEVKACSSELESLEMELRQQILVNIGKILQDQPS<br>MEALEASLGQGLCSGGQVEPLDGPAGCILECLVLDSGELVPELAAPIFYLL<br>GALAVLSETQQQLLAKALETTVLSKQLELVKHVLEQSTPWQEQSSVSLPT<br>VLLGDCWDEKNPTWVLLEECGLRLQVESPQVHWEPTSLIPTSALYASLFL<br>LSSLGQKPC
nFKBP-GD-CT-Flag	MGSSKSKPKDPSQRRSGVQVETISPGDGRTFPKRGQTCVVHYTGMLED<br>GKKVDSSRDRNKPFKFMLGKQEVIRGWEEGVAQMSVGQRAKLTISPDYA<br>YGATGHPGIIPPHATLVFDVELLKLETRGVQVETISPGDGRTFPKRGQTCV<br>VHYTGMLEDGKKVDSSRDRNKPFKFMLGKQEVIRGWEEGVAQMSVGQR<br>AKLTISPDYAYGATGHPGIIPPHATLVFDVELLKLEEFGIDEEELIEAADFQGL<br>YAEVKACSSELESLEMELRQQILVNIGKILQDQPSMEALEASLGQGLCSGG<br>QVEPLDGPAGCILECLVLDSGELVPELAAPIFYLLGALAVLSETQQQLLAKA<br>LETTVLSKQLELVKHVLEQSTPWQEQSSVSLPTVLLGDCWDEKNPTWVLL<br>EECGLRLQVESPQVHWEPTSLIPTSALYASLFLLSSLGQKPCDYKDDDDK<br>EFSESQ
cFKBP-GD-CT-Flag	MGSSKSKPKDPSQRRSEFGIDEEELIEAADFQGLYAEVKACSSELESLEM<br>ELRQQILVNIGKILQDQPSMEALEASLGQGLCSGGQVEPLDGPAGCILECL<br>VLDSGELVPELAAPIFYLLGALAVLSETQQQLLAKALETTVLSKQLELVKHV<br>LEQSTPWQEQSSVSLPTVLLGDCWDEKNPTWVLLEECGLRLQVESPQVH<br>WEPTSLIPTSALYASLFLLSSLGQKPCDYKDDDDKEFGVQVETISPGDGRT<br>FPKRGQTCVVHYTGMLEDGKKVDSSRDRNKPFKFMLGKQEVIRGWEEG<br>VAQMSVGQRAKLTISPDYAYGATGHPGIIPPHATLVFDVELLKLETRGVQVE<br>TISPGDGRTFPKRGQTCVVHYTGMLEDGKKVDSSRDRNKPFKFMLGKQE<br>VIRGWEEGVAQMSVGQRAKLTISPDYAYGATGHPGIIPPHATLVFDVELLKL<br>E
nmFlag-GD-FL	MDYKDDDDKGSPSAFEKVVKNVIKEVSGSRGDLIPVDSLRNSTSFRPYCL<br>LNRKFSSSRFWKPRYSCVNLSIKDILEPSAPEPEPECFGSFKVSDVVDGNI<br>QGRVMLSGMGEGKISGGAAVSDSSSASMNVCILRVTQKTWETMQHERH<br>LQQPENKILQQLRSRGDDLFVVTEVLQTKEEVQITEVHSQEGSGQFTLPG<br>ALCLKGEGKGHQSRKKMVTIPAGSILAFRVAQLLIGSKWDILLVSDEKQRTF<br>EPSSGDRKAVGQRHHGLNVLAALCSIGKQLSLLSDGIDDYKDDDDKGSGI<br>DEEELIEAADFQGLYAEVKACSSELESLEMELRQQILVNIGKILQDQPSMEA

	LEASLGQGLCSGGQVEPLDGPAGCILECLVLDSGELVPELAAPIFYLLGAL AVLSETQQQLLAKALETTVLSKQLELVKHVLEQSTPWQEQSSVSLPTVLLG DCWDEKNPTWVLLEECGLRLQVESPQVHWEPTSLIPTSALYASLFLLSSL GQKPCYPYDVPDYA
